# Toxic threats from plastic waste: human health impacts, challenges, and policy solutions

**DOI:** 10.1039/d5ra05845g

**Published:** 2025-10-27

**Authors:** Abdulaziz A. M. Abahussain, Fahd A. Nasr, Abdulrahman bin Jumah, P. Saravanan, Nadavala Siva Kumar, Mohammed Al-zharani, L. Guganathan, G. Sasikumar, Sulaiman A. Alsalamah, Ashraf Ahmed Qurtam, J. Senthilkumar, P. Tamizhdurai

**Affiliations:** a Chemical Engineering Department, College of Engineering, King Saud University P.O. Box 800 Riyadh 11421 Saudi Arabia; b Biology Department, College of Science, Imam Mohammad Ibn Saud Islamic University (IMSIU) Riyadh 11623 Saudi Arabia; c Department of Chemistry, St. Joseph's College of Engineering OMR Chennai 600119 India; d Department of Physics, Saveetha School of Engineering, Saveetha Insitute of Medical and Technical Science (SIMATS) Thandalam Chennai 602105 India; e Department of Chemistry, St. Joseph's College of Engineering Semmencherry, OMR Chennai Tamil Nadu India; f School of Mechanical Engineering, Sathyabama Institute of Science and Technology Chennai 600 119 India; g Department of Chemistry, Dwaraka Doss Goverdhan Doss Vaishnav College 833, Gokul Bagh, E.V.R. Periyar Road, Arumbakkam Chennai 600 106 Tamil Nadu India tamizhvkt2010@gmail.com

## Abstract

Plastic pollution has escalated into a global crisis, undermining both environmental sustainability and public health. Each year, nearly eight billion tons of plastic enter aquatic ecosystems, disrupting marine biodiversity and increasing risks of human exposure to toxic byproducts. As plastics degrade, they release hazardous compounds such as bisphenol A (BPA), dioxins, phthalates, furans, and heavy metals—substances linked to respiratory illnesses, endocrine disruption, and cancer. These pollutants find pathways into the environment through soil leaching, air transport, and bioaccumulation across the food chain. Alarming data points highlight the gravity of the issue: BPA concentrations in freshwater bodies have exceeded 12 μg L^−1^, and dioxin levels near open waste combustion sites have reached over 1000 ng TEQ per kg both surpassing WHO's safety thresholds. In Poland, phthalate levels in leachate from landfills have been recorded at more than 303 μg L^−1^, while fish specimens from Swedish waters have shown heavy metal concentrations more than 2.26 ng g^−1^ moist weight, raising potential food safety hazards and chronic exposure risks. Ineffective trash disposal infrastructure disproportionately affects vulnerable communities in countries with low to middle-incomes (LMICs), especially in Sub-Saharan Africa, where only 39–45% of solid waste is adequately treated due to infrastructural and financial limitations. The problem is most severe in Sub-Saharan Africa, where the most vulnerable people are at the greatest risk of exposure. On a global scale, effective recycling remains minimal, with less than 10% of plastic waste being repurposed. Most of it is incinerated or dumped, releasing hazardous emissions such as dioxins and furans into the environment. Emerging technologies offer promising but underutilized solutions. Polyethylene terephthalate (PET) can be recovered up to 97% using chemical recycling processes and enzymatic procedures have demonstrated plastic breakdown efficiencies as high as 90% within hours. However, challenges remain in scaling these technologies due to high costs, limited infrastructure, and uneven access in LMICs. Policy interventions have shown strong results in some nations. By enforcing restrictions on single-use plastics and applying expanded producer responsibility (EPR) frameworks, countries such as Germany, Japan, and Rwanda have achieved recycling rates above 41% and reduced plastic waste generation by up to 90%. These cases demonstrate the role of governance, regulatory enforcement, and accountability in driving systemic change. Addressing plastic pollution globally requires coordinated action, including strict regulations, investment in scalable recycling systems, promotion of eco-friendly alternatives, and stronger international cooperation. Only through such combined and multi-dimensional approaches can the growing environmental and public health risks of plastic waste be effectively reduced.

## Abstract 2

Plastic pollution is a global crisis, harming ecosystems and human health. Plastics release toxic compounds like BPA, dioxins, and heavy metals, bioaccumulate in food chains, and persist due to poor waste management, especially in low-income regions.

## Graphical abstract

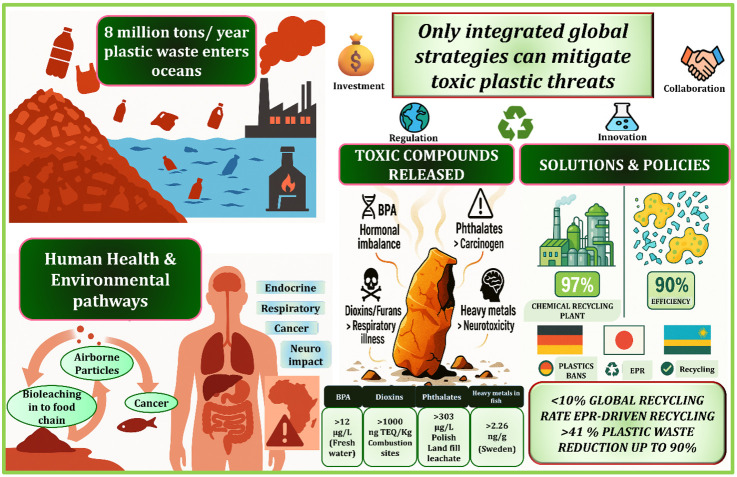

## 1. Introduction

A significant worldwide concern that poses grave risks to both human health and environmental sustainability is the growing amount of plastic garbage.^1^ Driven by the rapid growth of plastic production and insufficient waste management, pollution levels have increased sharply over the past few decades.^2^ The annual entry of over eight million tonnes of plastic debris into the oceans accelerates marine degradation, disrupts aquatic ecosystems, and contributes to the formation and spread of microplastics.^3^ The detrimental impacts of plastic trash have been extensively studied, especially with regard to the production and dissemination of microplastics. However, most existing research remains focused on environmental impacts, with comparatively little attention paid to the broader implications for human health.^4,5^

Emerging evidence highlights that plastic pollution is not only an ecological issue but also a direct human health concern. During production, use, and degradation, plastics release toxic compounds such as BPA, phthalates, dioxins, furans, and heavy metals.^6–8^ These chemicals are recognized for their ability to interfere with hormonal systems and contribute to chronic illnesses. Scientific studies have increasingly associated exposure to such pollutants with disruptions in endocrine function, developmental abnormalities, reproductive disorders, immune system impairment, and a heightened risk of certain cancers, underscoring the urgent need for public health intervention and stricter environmental safeguards.^9^ Contamination has been documented in soil, water, and air, raising concerns about multiple exposure routes. Particularly vulnerable populations including infants and pregnant women—may face disproportionate risks. Yet, the pathways of exposure remain insufficiently understood, and the combined or synergistic effects of multiple plastic-derived chemicals are rarely studied, leaving critical gaps in knowledge.^10,11^

Despite growing recognition of these hazards, current mitigation strategies have notable shortcomings. While efforts such as recycling and improved waste management have been promoted to reduce plastic pollution, these initiatives often emphasize environmental protection over direct health outcomes.^12^ Moreover, technological constraints such as incomplete detoxification during recycling reduce their effectiveness. In many low- and middle-income countries (LMICs), weak waste treatment infrastructure further undermines plastic waste management, exacerbating both environmental and health risks.^13^

While various technological innovations have been explored to tackle plastic pollution, policy-based strategies are also gaining attention for their potential to curb the production and use of harmful plastics. Measures such as bans on specific plastic categories and extended producer responsibility (EPR) programs have been proposed as implements for cutting down on plastic trash (US-EPA, 2024a).^14^ Nevertheless, implementation remains inconsistent due to weak institutional capacity, insufficient regulatory enforcement, and the lack of global consensus on policy models.^15^ This fragmented governance framework has limited progress toward effective plastic pollution management.

This paper reviews how toxins from plastic waste enter human and ecological systems, the health hazards linked to such exposures, and the current range of mitigation and policy responses. By structuring the review across environmental impacts, health risks, and governance challenges, it identifies critical research gaps and limitations in prevailing strategies. The objective is to provide a clearer foundation for developing integrated solutions that simultaneously address environmental degradation and public health risks, guiding stakeholders toward more coordinated and effective plastic waste management strategies.^16^

This review is structured around three dimensions environmental impacts, human health risks, and governance challenges to provide an integrated understanding of plastic-derived toxins. It examines key pollutants including bisphenol A, phthalates, dioxins, heavy metals, and micro- and nanoplastics, with emphasis on their toxicological effects, environmental fate, and bioaccumulation. In addition, it highlights barriers in waste management, recycling, and regulatory systems while assessing emerging technological and policy solutions. By identifying critical research gaps and inequities in exposure, particularly in low-income regions, this review aims to inform coordinated strategies that safeguard both ecological sustainability and public health.

## 2. Toxins in plastics: environmental fate, bioaccumulation, and human exposure

### 2.1. Toxicological profile of bisphenol A and phthalates: sources, exposure, and effects

Phthalates and bisphenol A (BPA) are synthetic compounds extensively used in plastic manufacturing, and their uncontrolled release has emerged as a significant ecological and health concern. Phthalates function primarily as plasticizers substances incorporated into plastics such as polyvinyl chloride (PVC) to improve flexibility, softness, and durability.^17^ They are commonly found in products including food packaging, cosmetics, adhesives, medical tubing, and children's toys. In contrast, BPA is an essential component in the manufacture of polycarbonate plastics and epoxy resins, widely applied in hard plastic drinkware, food storage containers, and the inner coatings of metal cans.^18^

A key issue is that neither BPA nor phthalates are covalently bonded to the polymer matrix. Because these additives are not covalently bonded to the polymer structure, they can gradually migrate out of the material and contaminate surrounding environments.^19^ This process is accelerated by external factors such as heat exposure, ultraviolet radiation, or mechanical stress.^20^ As these compounds leach out, they can enter air, water, soil, and even human bodies, raising concerns due to their established links with hormonal imbalance, reproductive toxicity, and other adverse health outcomes.^21,22^

Recent studies indicate that microplastics and nanoplastics act as carriers for BPA and phthalates, adsorbing these chemicals onto their surfaces and facilitating long-distance transport in marine and terrestrial ecosystems. This interaction increases the persistence and distribution range of such toxins beyond their immediate source.

These observations set the stage for understanding the direct toxicological impacts on human health, which are discussed in the following section.

### 2.2. Toxicological impacts of BPA and phthalates on human health

Building on their environmental release, these compounds can reach humans through multiple exposure pathways. In addition to direct leaching from consumer products, open burning and inefficient incineration of plastic waste release these compounds along with heavy metals (Pb, Cd, Hg), volatile organic compounds (VOCs), fine particulate matter (PM), and polycyclic aromatic hydrocarbons (PAH).^23–25^ More persistent organic pollutants such as polychlorinated biphenyl (PCB), dioxins, and furans accumulate in the food chain, causing cancers, immune dysfunction, and developmental issues.^26^ Safer waste management and stricter regulations are urgently needed.

**Fig. 1:**
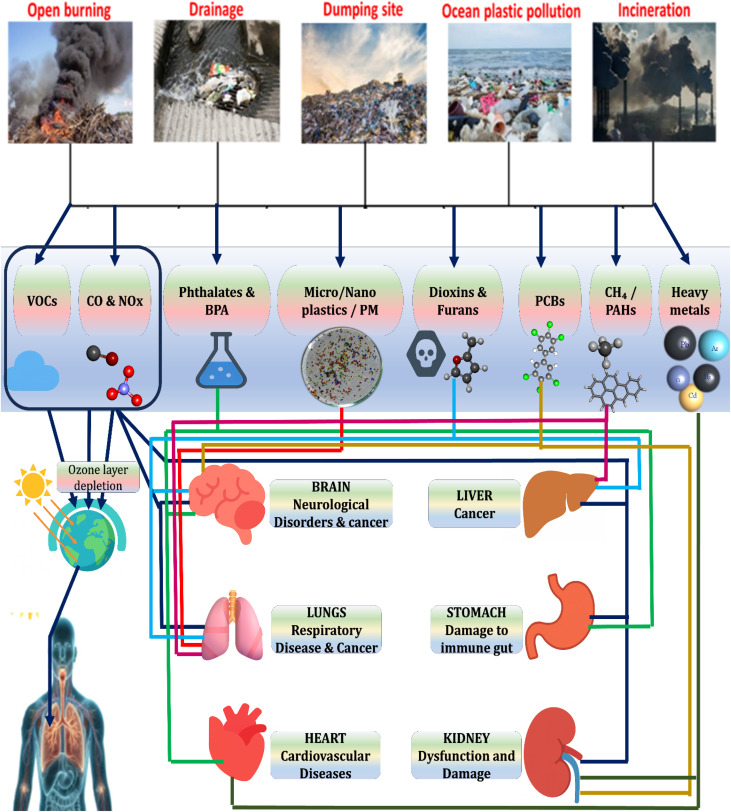
Toxic trails: health impacts of plastic pollution.^27^

Fig. 1 illustrates the connections between environmental contamination and adverse health outcomes, emphasizing the multi-systemic effects of these substances. Once introduced into the environment, these pollutants contribute to ecosystem degradation and pose substantial risks to human health. They can adversely affect various organ systems, including the nervous, respiratory, cardiovascular, reproductive, and digestive systems. Fig. 1 further highlights specific health conditions linked to these toxins, such as hormonal disruptions, respiratory illnesses, cognitive impairments, cardiovascular diseases, and gastrointestinal disorders. Additionally, the formation of ground-level ozone from VOCs, carbon monoxide (CO), and nitrogen oxides (NO_*x*_) amplifies the health risks, particularly among sensitive populations.^28^

Phthalates and BPA are of particular concern due to their extensive environmental prevalence and capacity to interfere with hormonal regulation. Fig. 1 illustrates how these substances, once released, can enter biological systems and cause harmful physiological effects.^29^ These compounds mimic natural hormones such as estrogen, disrupting normal endocrine signaling. Research has shown that early-life exposure to phthalates may impair sperm quality, disrupt male reproductive development, and reduce testosterone levels. An elevated incidence of reproductive malignancies and alterations in the onset of puberty have been linked to such exposure in females. Likewise, BPA has been connected to metabolic diseases like unhealthy weight and type 2 diabetes, neurological abnormalities, and breast and prostate carcinomas.^30^

These health effects are especially pronounced in vulnerable groups such as foetuses, infants, and young children, whose developing hormonal systems make them more susceptible to endocrine disruption.^31^ Scientific studies indicate that exposure during critical developmental stages may result in lasting consequences, including cognitive deficits, weakened immune responses, and elevated risk of cardiovascular disease. Due to the persistent use of these chemicals in everyday consumer and industrial products, exposure remains widespread, underscoring a prevalent globe-wide healthcare concern.^32^

### 2.3. Phthalates & BPA: long-term impact and bioaccumulation

The extensive application of BPA and phthalates in the processing of plastics has led to serious environmental and public health issues owing to its longevity and mobility. Because these compounds are not covalently bonded to plastics, they continuously leach into the environment under various conditions.^33^ Phthalates are commonly found in garbage dumps discharges as well as aquatic systems, with some sites reporting levels surpassing 303 μg L^−1^.^34^ Phthalates serve for enhancing the flexibility of plastic components like polyvinyl chloride (PVC). Commonly found in epoxy-based coatings and polycarbonate plastics, BPA has also been found in many water bodies around the world; in certain Chinese waterways, concentrations have been as high as 12 μg L^−1^. After entering the environment, both phthalates and BPA accumulate in soil, sediments, and water and can travel far from their original sources, creating long-lasting ecological and toxicological threats.^34^

The environmental and physiological effects of BPA and phthalates are further increased by their ability to accumulate in cells and tissues. Endocrine-disrupting chemicals (EDCs) are substances that can interfere with hormonal systems by either blocking or mimicking the natural hormone activity.^35^ Humans are primarily exposed to microplastics through ingestion of contaminated food and water. Other possible pathways include inhalation of airborne particles and dermal absorption through contact. Research conducted in North America and Europe has reported elevated levels of phthalates and BPA in aquatic life, including mollusks, crustaceans, and fish, with higher concentrations often observed at higher trophic levels. Studies conducted in the Northeast Atlantic have found that fish tissues contain as high as 300 ng g^−1^ wet weight of BPA, which raises worries about the possibility of human exposure from eating seafood.^36^ Phthalates have also been detected in crops irrigated with contaminated water, particularly in regions with intensive farming and poor water quality control, such as parts of China and Mexico.^37^

Repeated or heated use of plastic food containers further increases the risk of human exposure.^38^ EU studies showed that over 10% of polycarbonate containers tested leached BPA at levels above the safety threshold of 0.05 mg kg^−1^. Even minimal, long-term exposure to these substances has been associated with serious health concerns, including hormonal imbalances, fertility issues, and developmental abnormalities. Pregnant women, infants, and young children are particularly vulnerable due to critical developmental stages.^39^

Bioaccumulation refers to the progressive build-up of substances, such as microplastics (MPs) and associated chemicals, within an organism when uptake exceeds elimination. In plastics, this process may be compounded by their ability to adsorb and transport toxic additives or pollutants. Biomagnification, by contrast, describes the increasing concentration of MPs and associated chemicals across successive trophic levels. Current evidence shows that MPs bioaccumulate within marine species, but there is little support for biomagnification under environmentally realistic conditions. Moreover, data on the accumulation and trophic transfer of chemical additives remain limited and ambiguous, highlighting the need for studies on ingestion, retention, and depuration processes.^40^

### 2.4. Dioxins and furans: sources, health impacts, and environmental fate

Dioxins and furans are among the most hazardous pollutants released during the burning of plastic materials, especially under poorly controlled or low-temperature combustion conditions. These compounds are generated during thermal operations between 200 °C and 800 °C, with peak formation typically occurring between 350 °C and 400 °C. Extremely resilient compounds include 2,3,7,8-tetrachlorodibenzo-*p*-dioxin (TCDD), allowing them to remain in the environment for extended periods without significant breakdown.^41^ Plastics that contain chlorine such as polyvinyl chloride (PVC) play a significant role in their generation. During the combustion of chlorinated polymers, chlorine interacts with carbon-based compounds, resulting in the creation of these toxic by-products.^42^ This phenomenon is especially common in regions where waste is openly burned, such as landfills or informal dumpsites, which are frequently encountered in developing areas with limited access to formal waste disposal systems (WHO, 2024).^43^

Dioxins and furans are classified as persistent organic pollutants (POPs) due to their resistance to degradation and long environmental half-lives, ranging from 7 to 11 years in soil. After emission, these compounds can be transported over vast distances before settling onto land, water, and plant surfaces, ultimately infiltrating the food web.^44^ Soil dioxin levels exceeding 1000 ng TEQ per kg have been reported in regions practicing open burning, such as Sweden, surpassing WHO safety thresholds. Similarly, air near informal electronic waste dismantling sites in Ghana has shown dioxin-like PCB concentrations of 280–11,100 pg m^−3^.^45^

Because dioxins and furans are lipophilic, they bioaccumulate in fatty tissues of animals, becoming more concentrated as they move up the food chain.^46^ Marine environments are especially under risk. Dioxin levels in edible fish fat have been shown to reach 150 picograms/gram in Japanese research, raising concerns about the safety of seafood for populations that rely heavily on marine food sources. Even remote regions like the Arctic are not spared traces of dioxins have been found in the fat of seals and polar bears, carried to these distant environments through atmospheric transport. This global spread highlights the far-reaching threat dioxins and furans pose to both ecosystems and human health, regardless of proximity to pollution sources.^47^

Exposure pathways for humans include ingestion of contaminated food, inhalation of polluted air, and dermal contact with contaminated soils or dust. These compounds are highly toxic even at low concentrations (1–10 ppt in blood, ∼15 ppt in serum lipids) and bind to the aryl hydrocarbon receptor (AhR), disrupting gene expression and cellular function. As seen in Fig. 1, major sources include waste incineration, uncontrolled burning of materials, and various industrial processes. Once released, dioxins and furans contaminate air, soil, and water, where they persist and bioaccumulate.^48^ Health impacts include chloracne, immune suppression, liver toxicity, endocrine disruption (particularly thyroid dysfunction), reproductive and developmental toxicity, congenital malformations, developmental delays in children, miscarriage, premature birth, restricted fetal growth, and an increased risk of cancers.^49–52^

The public health threat is especially severe in regions with inadequate waste management, such as India and Nigeria, where open burning of municipal and electronic waste is common. In contrast, countries like Sweden, France, and Germany have reduced emissions through advanced incineration technologies with effective filtration systems. However, many low- and middle-income nations lack the financial and technical capacity to implement such systems.^53^

### 2.5. Mechanistic pathway of dioxin and furan formation from plastics

The formation of polychlorinated dibenzo-*p*-dioxins (PCDDs) and polychlorinated dibenzofurans (PCDFs) during thermal treatment of plastics, particularly polyvinyl chloride (PVC), has been widely studied. The process occurs through a series of thermal degradation, radical-driven reactions, aromatic condensation, and oxidative coupling steps.^54,55^

Step 1: thermal decomposition of PVC: at 200–800 °C, PVC undergoes pyrolysis:
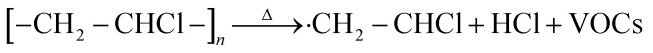


This process liberates vinyl radicals, chlorine radicals, hydrogen chloride (HCl), and volatile organic compounds (VOCs). The release of HCl plays a dual role: promoting chlorination reactions and acting as a catalyst for further degradation.

Step 2: radical formation: fragmentation of vinyl chloride generates carbon-centered radicals:
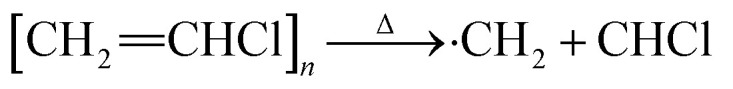


These radicals are highly reactive intermediates that initiate condensation and substitution reactions.

Step 3: condensation and cyclization: reactive radicals recombine, producing aromatic rings and PAH:˙CH=CH → C_6_H_6_(benzene)

Subsequent chlorination of aromatics yields chlorobenzenes:
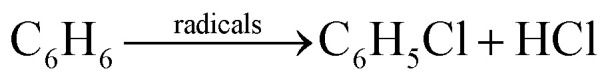


These chlorinated aromatics serve as critical precursors to PCDDs and PCDFs.

Step 4: oxidative coupling: in oxygen-rich conditions, chlorophenoxy radicals undergo oxidative coupling:

For dioxins:2C_6_H_4_ClO˙ + O_2_ → C_12_H_4_ClO_2_(dioxin)

For furans:C_12_H_4_ClO_2_˙ → C_12_H_4_Cl_4_O(furan)

This step forms the PCDD and PCDF core structures, including the toxic congener 2,3,7,8-TCDD.

Step 5: stabilization: once formed, PCDD/F congeners, such as TCDD, exhibit high thermodynamic stability due to their conjugated aromatic structures:C_12_H_4_Cl_4_O ↛ further degradation

This stability underpins their persistence in environmental matrices.

Step 6: environmental release: PCDDs and PCDFs are released into the atmosphere and subsequently deposited onto soil and water surfaces. Their lipophilicity promotes bioaccumulation in fatty tissues, enabling entry and magnification through the food chain.

### 2.6. Environmental and health hazards of heavy metals

Heavy metals present in plastic waste pose serious risks to both environmental quality and public health due to their inherent toxicity, long-term persistence, and ability to bioaccumulate in ecosystems. Commonly used metals such as lead (Pb), cadmium (Cd), chromium (Cr), and mercury (Hg) are often added during plastic production to improve physical properties, thermal stability, and color. For example, lead has traditionally been used in polyvinyl chloride (PVC) to enhance durability, while cadmium imparts bright coloration to plastic products.^56^ Mercury may be present in specific stabilizers and catalytic agents, and hexavalent chromium is utilized in dyes to produce distinct color tones. Although these additives contribute to the functionality and appearance of plastic goods, they also increase the materials' resistance to degradation. When improperly disposed of whether through open burning, poorly managed landfills, or inefficient incineration these plastics release heavy metals into surrounding environments, leading to long-lasting contamination and associated health hazards.^57^ Heavy metals can increase the resistance of plastics to degradation in several ways. Certain metals (*e.g.*, cadmium, lead, chromium) are used as stabilizers, pigments, or catalysts in polymer formulations, where they inhibit photodegradation and thermal oxidation by quenching free radicals or altering polymer chain chemistry. In addition, the incorporation of metal-based additives can modify the crystallinity and density of polymers, reducing their susceptibility to microbial and enzymatic attack. As a result, plastics containing heavy metal additives often persist longer in the environment compared to untreated counterparts.^58^

### 2.7. Heavy metal hazards: public health threats and policy barriers

Exposure to heavy metals originating from plastic waste presents significant health challenges, particularly in areas where proper waste management systems are absent. Lead is one of the most concerning elements due to its irreversible effects on the nervous system. Children are especially at risk, with exposure linked to cognitive decline, behavioural issues, and impaired learning. In cities across Nigeria, informal recycling operations have been associated with elevated lead levels in children living nearby, indicating a critical public health concern. Cadmium is another hazardous element commonly found in plastic-related activities.^59^ Long-term exposure has been connected to kidney damage, bone fragility, and respiratory complications. Studies conducted in Bangladesh have observed increased cadmium concentrations in the urine of individuals living close to plastic production and recycling zones, with many also reporting kidney ailments. Mercury, released during the burning of plastic waste, poses serious neurological risks.^60^ Open-air incineration practices in parts of India contribute to rising mercury emissions, raising alarms over the impact on both children's and adults' brain health and nerve function. In Ghana, particularly around the Agbogbloshie site—one of Africa's largest informal e-waste and plastic recycling centers—workers have been found to exhibit extremely high blood lead levels, often exceeding the WHO guideline of 5 μg dL^−1^ for children.^61,62^ Soil and dust in the area contain lead concentrations far above international safety limits, surpassing the U.S. EPA maximum contaminant level (MCL) of 400 ppm in soil.^63^ These findings underscore the urgent need for robust environmental safeguards, regulated disposal systems, and targeted public health initiatives, as unregulated recycling and open burning of waste place entire communities at risk of chronic lead exposure and its associated health impacts, including neurodevelopmental deficits in children.^64^

### 2.8. Heavy metal leaching from plastics: environmental consequences and concerns

Improper methods of plastic trash disposal include open dumping or else unrestrained incineration pose a significant environmental risk due to the potential release of toxic heavy metals. As plastic materials degrade under environmental conditions like sunlight, mechanical friction, and moisture, embedded additives, including heavy metals, can slowly leach into surrounding ecosystems.^65^ This is especially true for plastics like polyvinyl chloride (PVC), which often contain lead (Pb) and cadmium (Cd) as stabilizing agents. When these materials are placed in landfills, they can persist for decades. Heavy metals have the potential to move into the local groundwater and soil during this period, which might have an impact on drinking water supplies and agricultural areas. Studies indicate that even minimal concentrations of lead around 10 parts per million can disrupt soil chemistry and suppress plant growth. Cadmium, similarly, tends to accumulate in crops cultivated near contaminated zones, raising concerns about food chain contamination and dietary exposure to harmful substances.^66^

The environmental threat intensifies when plastic waste is incinerated without adequate emission controls. Burning plastics like PVC can release heavy metals into the air, either attached to particulate matter or in vapor form. These emissions may pose serious health risks to communities located near such burn sites. Research links this practice to the atmospheric presence of not only lead and cadmium but also mercury (Hg) and arsenic (As), all of which are known for their toxicological impacts on humans. One of the most concerning aspects of heavy metal pollution is its persistence.^67^ Unlike many organic compounds, metals such as Cd, Hg, and Pb, never decompose over time. In aquatic environments, they may settle in sediments or enter biological systems, leading to accumulation in organisms. Through the process of biomagnification, these contaminants can become increasingly concentrated as they move up the food chain eventually reaching individuals those consume seafoods from polluted waters.^68^

### 2.9. Micro- and nanoplastics: hidden contaminants in ecosystems

Larger plastic materials gradually degrade when subjected to natural factors like heat, sunlight, and mechanical abrasion, producing microplastics and nanoplastics as pollutants. Unlike biodegradable matter, plastics fragment into progressively smaller pieces rather than decomposing, yielding microplastics (typically <5 mm) and nanoplastics (often <100 nm). This fragmentation process can take decades, during which the particles remain in the environment and may be absorbed by plants, animals, and humans. Their widespread distribution in the atmosphere, water, soils and food chain has sparked serious concerns about their potential hazards to public health and habitats.^69^

Recent systematic assessments, including those by the European Food Safety Authority (EFSA), have highlighted that although microplastics are ubiquitously detected across ecosystems, standardized sampling and analytical methods remain inconsistent, which complicates efforts to compare results across regions and to establish reliable global baselines.^70^

### 2.10. human exposure pathways and toxicological insights

Micro- and nanoplastics (MNPs) pollution has become a pressing environmental and public health concern. Humans are exposed through oral ingestion, inhalation, and dermal contact, with sources ranging from contaminated food and beverages to indoor dust and industrial air.^71–73^ The COVID-19 pandemic further exacerbated plastic pollution due to widespread use of PPE and single-use plastics, which contribute additional MPs and NPs to the environment [Alqahtani].

Experimental evidence from *in vivo* and *in vitro* models demonstrates that MNPs can induce oxidative stress, cytotoxicity, immune dysregulation, endocrine disruption, neurotoxicity, reproductive and developmental toxicity, and organ dysfunction.^71,72,74^ Small MPs and NPs can translocate from the gastrointestinal tract and lungs into secondary tissues, whereas dermal absorption is generally limited.^80^ Epidemiological data are limited, with only preliminary associations reported for conditions such as asthma, lung nodules, and blood thrombus, highlighting critical uncertainty regarding chronic exposure, bioavailability, and dose–response relationships.^71,72^

Ecologically, MNPs serve as vectors for persistent organic pollutants (POPs) such as PCBs, PAHs, and legacy pesticides, promoting bioaccumulation and biomagnification in food webs [EFSA 2025]. Soil and marine ecosystems are affected as microplastics alter soil structure, water retention, and feeding efficiency of aquatic organisms, potentially impacting biodiversity and ecosystem stability. Critical reviews underscore that ecological risk assessments remain limited by the lack of long-term field data, particularly under interacting climate stressors like ocean acidification and rising temperatures.^76^

Recent systematic reviews of Feng *et al.*, 2023; Ramsperger *et al.*, 2023; Alqahtani *et al.*, 2023 support these mechanistic observations yet highlight the scarcity of quantitative data on human exposure.^72–74^ These works call for harmonized methodologies, integrative toxicological and epidemiological studies, and standardized reporting of particle size, composition, and exposure scenarios to reduce uncertainty and enable robust risk assessment.

### 2.11. Health hazards associated with micro- and nanoplastics

Microplastics, due to their extremely small size and complex chemical composition, can enter the human body and interact with internal systems in harmful ways. Once ingested, these particles may move beyond the digestive tract and reach other parts of the body through the bloodstream or lymphatic system. Their presence can trigger inflammatory responses, oxidative stress, and disrupt normal cell activity. Nanoplastics—particles even smaller than microplastics—are especially concerning because they can cross critical biological constraints including the blood–brain barrier and the gut lining. Research in South Korea, for example, has linked nanoplastic exposure to brain inflammation and impaired cognitive function in animal studies. In addition to their harmful physical consequences, microplastics frequently contain heavy metals, phthalates, BPA, and other endocrine disruptors, as well as persistent organic pollutants (POPs). These substances may seep from the plastic's surface, increasing the risk of reproductive problems, hormonal abnormalities, and cancer.^77–79^

**Fig. 2:**
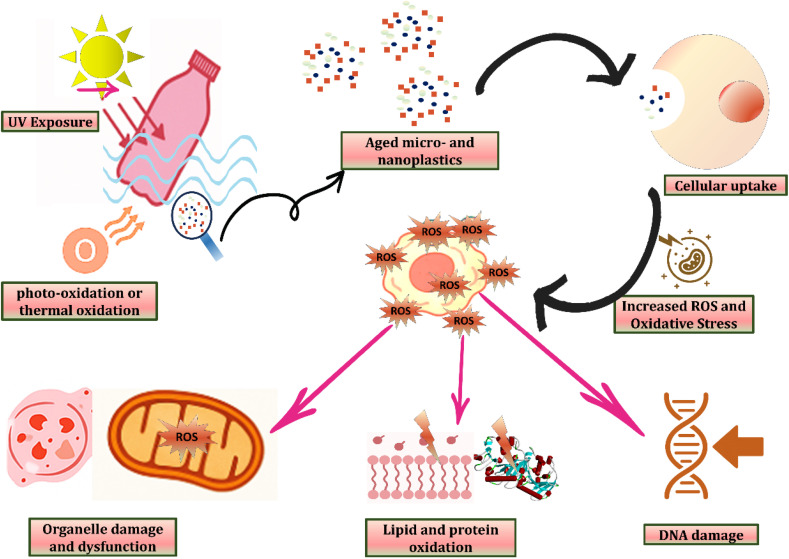
Schematic representation of the oxidative stress pathway induced by aged micro- and nanoplastics (MNPs).^80^

Environmental weathering processes such as UV irradiation, photo-oxidation, and thermal degradation promote the fragmentation of plastics into aged micro- and nanoplastics (MNPs). These smaller particles are highly reactive and can readily enter biological systems through ingestion, inhalation, and dermal penetration. Once internalized *via* cellular uptake, MNPs disrupt cellular redox balance, leading to the excessive formation of reactive oxygen species (ROS). Elevated ROS levels induce oxidative stress, initiating a cascade of toxicological effects. At the subcellular level, ROS compromise the structural integrity and function of organelles such as mitochondria, resulting in impaired energy metabolism and cellular dysfunction. Concurrently, oxidative modifications of lipids and proteins impair membrane stability and enzyme activity. Moreover, ROS-mediated DNA damage—including strand breaks and mutagenic alterations—poses risks for genomic instability, inflammation, and long-term health consequences such as carcinogenesis. Collectively, this pathway highlights the mechanistic link between environmental degradation of plastics and their biological toxicity, underscoring the urgent need for further research into human and ecological health risks posed by aged MNPs (Fig. 2).

Inhalation represents another exposure route. Airborne microplastics are especially prevalent in cities and industrial zones. Reported concentrations vary widely by region: studies have measured approximately 2 particles per m^2^ in Japan, 13 in Nepal, 20 in Vietnam (for particles >100 μm, excluding fibers), and up to 294 particles per m^2^ in certain Iranian regions (for particles >50 μm).^81^ These particles can irritate and inflame the respiratory system after being inhaled. In more extreme situations, they might penetrate lung tissues and aggravate long-term respiratory disorders. Occupational exposure is becoming a bigger issue, especially for workers in sectors like plastic waste removal and textile manufacture. While scientific understanding of micro- and nanoplastic exposure continues to grow, the full extent of their long-term health effects remains uncertain and warrants further investigation.^82^

Accurately quantifying micro- and especially nanoplastics in human tissues remains challenging due to limitations in current detection methods (*e.g.*, Raman spectroscopy, FTIR, electron microscopy), potential contamination or particle loss during sample preparation, and insufficient sensitivity for nanoscale particles, contributing to uncertainty about human bioavailability and chronic exposure risks.^71–75^

The World Health Organization (WHO) emphasizes that there is currently insufficient evidence to quantify the risks of chronic microplastic exposure in humans, particularly with respect to bioavailability and dose–response relationships, underscoring the urgent need for harmonized toxicological studies.^71^

### 2.12. Ecological threats posed by microplastics and nanoplastics

Microplastics and nanoplastics pose a serious environmental challenge due to their capacity to attract and transport harmful pollutants, particularly persistent organic pollutants (POPs). These pollutants—including substances such as PCB, PAH, and older pesticides like dichlorodiphenyltrichloroethane (DDT)—are known for their resistance to degradation and their tendency to accumulate in living organisms, leading to prolonged ecological and health impacts. Their small size, high surface area in relation to volume, as well as hydrophobic properties, microplastics readily bind with such contaminants, especially in aquatic systems. This allows them to act as mobile carriers, transporting pollutants through rivers, oceans, and other ecosystems.^65^

**Fig. 3:**
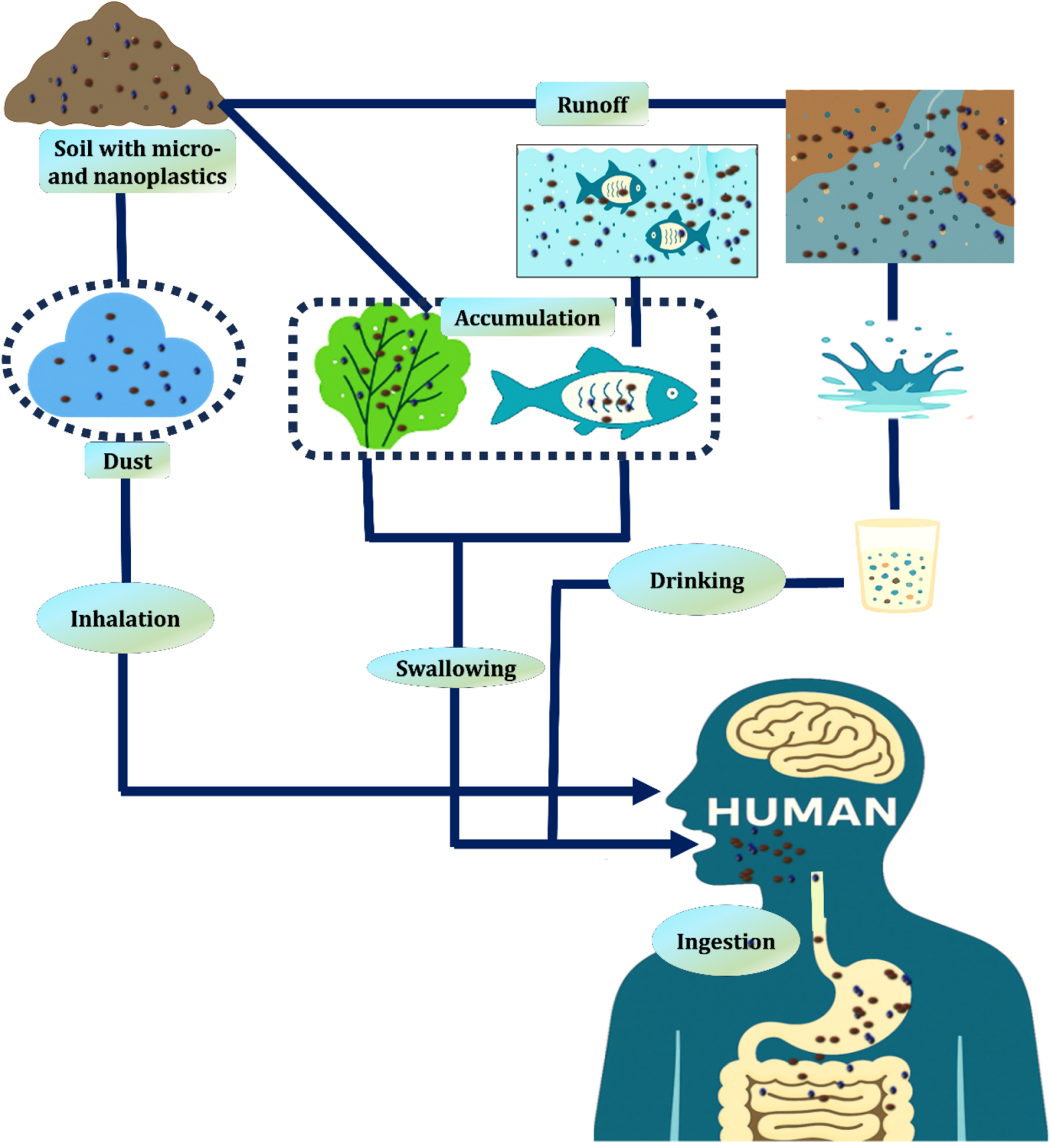
Humans are primarily exposed to micro- and nanoplastics through inhalation, ingestion of dust, and consumption of contaminated food and water, leading to their buildup in the human body.^84^

As illustrated in Figure.3 air, land, and water are contaminated by microplastics and nanoplastics that enter the environment through a variety of sources. Thereafter, they become ingrained in food chains after being eaten by a variety of creatures, including marine mammals and plankton.^83^ Ingested plastics carry harmful chemicals into animal tissues, initiating bioaccumulation, where toxic substances build up within individuals over time, and biomagnification, which causes these toxins to intensify at higher levels of the food web. Therefore, the greatest exposure dangers are faced by apex predators, such as humans. People come into contact with these contaminants primarily by eating polluted seafood, drinking tainted water, or breathing in microplastic particles from dust and synthetic materials.^85^

In addition to chemical hazards, microplastics and nanoplastics cause physical and ecological disturbances. In soil, they can alter properties such as water retention and structure, which may impair plant growth. In marine settings, filter feeders like mussels and small crustaceans may suffer reduced feeding efficiency when exposed to plastic particles, potentially impacting reproduction and survival. Research has found that blue mussels exposed to microplastics exhibit a noticeable decline in feeding activity, with implications for broader marine food networks. Furthermore, these plastic particles can aid the dispersal of invasive species by providing surfaces for attachment, thereby threatening local biodiversity and ecosystem stability.^86^

Critical reviews suggest that ecological risk assessments are still hampered by a lack of long-term field data, particularly regarding how micro- and nanoplastics interact with climate stressors such as ocean acidification and rising temperatures, which may amplify their ecological impacts.^87,88^

## 3. Barriers to reducing toxic emissions from plastic waste

### 3.1. Gaps in waste management systems

Lack of efficient waste management infrastructure is a major obstacle to addressing harmful plastic waste, especially in low- and middle-income nations. In many of these regions, the lack of organized collection and disposal systems leads to environmentally damaging practices such as open dumping and uncontrolled burning both of which release hazardous pollutants into the air, soil, and water. According to World Bank data, an alarming 93% of waste in low-income nations is either left uncollected or improperly discarded, in stark contrast to the more structured and regulated systems of high-income countries.^89^ This gap highlights the more profound differences in waste management capabilities across the two extremes of the globe are shown in Table 1. While wealthier nations often have access to advanced waste processing technologies, many developing regions struggle with basic collection and disposal. In parts of Sub-Saharan Africa, for example, cities in Nigeria, Kenya, and Liberia formally manage only 39–45% of their total waste. The remainder is frequently burned in open areas or dumped into the environment without oversight, contributing to significant ecological and public health hazards.^105^

**Table 1:** Contrasting plastic economies: resource allocation and waste strategies worldwide

S. No	Aspect	Developed countries	Developing countries	Ref
1	Annual plastic consumption (per capita)	∼100–130 kg per person per year (*e.g.*, USA: ∼130 kg; EU average: ∼110 kg)	∼10–35 kg per person per year (*e.g.*, India: ∼11 kg; Kenya: ∼25 kg)	90
2	Total plastic waste generated (2023)	USA: ∼42 million tons EU: ∼30 million tons Japan: ∼9 million tons	India: ∼9 million tons Nigeria: ∼3 million tons Indonesia: ∼7 million tons	91
3	Percentage of mismanaged plastic waste	<5% (*e.g.*, Germany: ∼1%, USA: ∼2%)	70–90% in low-income countries (*e.g.*, Nigeria: ∼88%, India: ∼75%)	92
4	Recycling rate (plastic waste)	30–45% (Germany: 46%, EU average: 41%)	<10% (India: ∼6%, Kenya: ∼7%)	93
5	Landfilling rate	10–30% (USA: ∼50% of plastic ends up in landfills)	60–90% or open dumping (India: ∼70%, Nigeria: ∼85%)	94
6	Waste-to-energy (incineration with energy recovery)	30–40% (Japan: ∼60%, Sweden: ∼50%)	<1% (due to lack of infrastructure and investment)	95
7	Presence of formal waste management infrastructure	High – mechanized collection, sorting, and processing systems in place	Low – informal sector dominates; manual collection and segregation	96
8	Budget allocation for waste management (avg.)	0.5–1.5% of GDP (*e.g.*, OECD countries)	<0.2–0.5% of GDP	97
9	Government regulation and enforcement	Strong regulatory frameworks (*e.g.*, EU REACH, US EPA, Japan's container and packaging recycling law)	Weak regulation and poor enforcement in most cases; limited data availability	98
10	Public awareness and participation	High – recycling bins, source segregation, deposit-return schemes	Low to moderate – limited education and awareness campaigns targeting schools, communities, and specific industries	99
11	Plastic import and export trends	Export of plastic waste to developing countries (declining post-basel amendments)	Major plastic waste importers until restrictions (*e.g.*, prior to China's national sword)	100
12	Use of alternatives (bioplastics, reusables)	Increasing adoption due to policy incentives and consumer demand	Low adoption due to high cost and limited availability and supply chain constraints	101
13	Challenges	Plastic overproduction, microplastic pollution, export dependency for recycling	Open burning, littering, lack of segregation, informal sector exploitation	102
14	Key initiatives	Extended producer responsibility (EPR), circular economy laws, plastic bans on specific items	Ban on single-use plastics (some countries), informal sector integration, international aid programs	103
15	Global impact (marine plastic leakage)	Contribute ∼2 million tons per year (mostly well-managed waste)	Contribute >80% of marine plastic leakage due to poor waste containment	104

In many urban centers across the Global South, waste systems are decentralized and heavily dependent on informal labour. Independent waste collectors and pickers who operate outside official frameworks are a vital part of the recycling ecosystem.^106^ However, they often work without protective equipment, formal training, or regulatory support, exposing themselves to hazardous substances embedded in plastics, such as BPA, phthalates, and hazardous heavy metals. Informal workers are essential to keeping rubbish out of landfills in nations like the Philippines and India, but they usually do so at significant personal danger and environmental cost.^107^

Another major concern is the lack of engineered landfills designed to contain plastic-related toxins. In many developing areas, disposal sites are either nonexistent or poorly managed, allowing contaminants like lead, mercury, and cadmium to leach into soil and water. Pakistan, for instance, has witnessed serious groundwater pollution due to unchecked plastic waste disposal, with long-term implications for agriculture, biodiversity, and human health. The absence of source-level waste segregation further exacerbates the problem, because hazardous plastics are frequently mixed with regular trash, treating or recycling them can be challenging.^108^

Even in high-income nations, where waste infrastructure is more advanced, challenges persist. Many recycling facilities are ill-equipped to process complex or chemically treated plastics. According to the OECD (2022a), plastics containing stabilizers or flame retardants especially multilayered materials often bypass recycling streams altogether. These substances are usually disposed of in landfills, where they might linger for generations, or burned, releasing pollutants including furans and dioxins. The surge in single-use plastic consumption globally has also begun to overwhelm even the most sophisticated waste systems, highlighting the urgent need for systemic reform and innovation in plastic management.^109^

### 3.2. Challenges in modern recycling methods

**Fig. 4:**
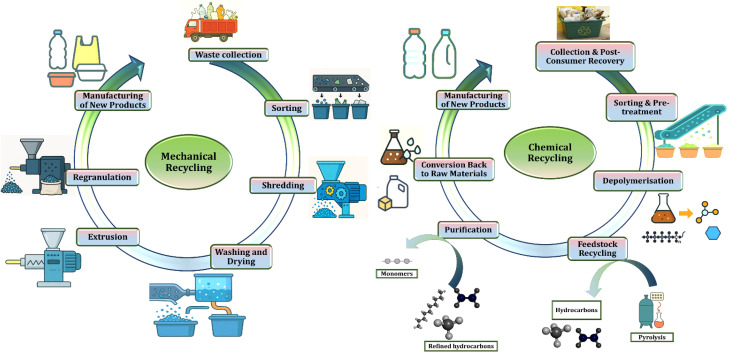
Schematic representation of mechanical and chemical recycling workflows, illustrating key steps involved in converting post-consumer plastic waste into new products.

Plastics can be recycled through mechanical and chemical routes. Mechanical recycling involves collection, sorting, washing, shredding, and reprocessing into pellets for manufacturing new products. In contrast, chemical recycling breaks plastics down at the molecular level *via* depolymerisation, pyrolysis, or gasification, producing monomers and hydrocarbons that can be purified and reused as raw materials. These complementary approaches are illustrated in Fig. 4, where mechanical recycling relies on physical reprocessing, while chemical recycling enables recovery of virgin-quality feedstocks from diverse waste streams.

Recycling is frequently marketed as an essential tactic to manage the plastic waste problem, yet existing methods have serious drawbacks that make it difficult to address the harmful risks connected to plastic pollution. Mechanical recycling, the most popular technique, entails gathering, classifying, and repurposing plastic waste to create new materials. While it appears to offer a practical means of reuse, this method has a major flaw: with each recycling cycle, plastic degrades in quality a phenomenon known as downcycling. As a result, the recycled output frequently fails to meet the stringent purity standards required for applications such as food packaging. Additionally, mechanical recycling does not remove hazardous chemicals commonly added to plastics, including bisphenol A, phthalates, and heavy metals. These substances remain in recycled materials and continue to pose risks to human health.^110^

Newer chemical recycling approaches such as depolymerization and pyrolysis attempt to overcome these limitations by breaking down plastics into their fundamental chemical components. While these methods offer the potential for producing higher-quality recycled materials, they are constrained by high energy use, complex infrastructure, and significant costs.^111^ As a result, these methods are currently only used to treat a small portion of the plastic garbage generated worldwide. In nations like India, where 9.5 million metric tons of plastic trash are produced each year, widespread adoption is slowed by limited energy access, inadequate technical capacity, and scarce financial resources. Furthermore, these processes can create additional environmental problems, such as toxic emissions and hazardous by-products, raising concerns about their sustainability. An added layer of complexity is presented by the increasing use of multi-layer and composite plastics in packaging.^112^ These materials often combine several types of polymers and may include metal foils or adhesives, making them difficult to separate and process. Existing recycling systems are generally not equipped to handle such complexity, leading to the routine disposal of these materials through incineration or landfilling. They both emit dangerous chemicals including hazardous metals and dioxins. Even in developed nations such as the United States, where annual plastic waste exceeds 35.7 million tons, overall recycling rates remain under 10%, with composite plastics being among the most difficult to recover.^113^

Contamination within recycling streams is another significant obstacle. Plastics contaminated by food residues, grease, or chemicals are often rejected by recycling facilities and instead diverted to incinerators or landfills. This not only reduces recycling efficiency but also increases the likelihood of toxic exposure during processing. In areas without reliable systems for separating waste common in many Southeast Asian regions contamination is widespread, limiting the effectiveness of recycling efforts and worsening the environmental and public health risks linked to plastic waste.^114^

### 3.3. Limitations in environmental regulation and compliance

Efforts to control plastic waste and its toxic components are hindered by a range of challenges, notably the absence of consistent regulations and weak enforcement across different regions. A unified global approach to managing hazardous chemicals found in plastics remains elusive. For instance, the European Union has set in place comprehensive frameworks like the RoHS Directive and REACH to limit the use of dangerous compounds.^115^ However, the degree of enforcement and compliance varies significantly among member states. However, many LMICs (low- and middle-income nations) have yet to develop comprehensive legislation targeting toxic elements in plastic waste. Data shows that only around 13% of African nations have adopted national laws focused on hazardous plastic materials, leaving large areas without effective regulatory coverage.

Even where laws do exist, enforcement is often compromised by a lack of financial resources, infrastructure, and technical expertise. In many LMICs, the informal sector plays a dominant role in waste management, frequently operating without oversight or safety protocols. For instance, in India, informal workers manage up to 90% of the country's waste, yet lack the capacity to safely identify or eliminate hazardous plastic additives. Regulatory shortcomings are not limited to developing countries. Recurrent infractions of hazardous waste restrictions are still being reported in the United States, with numerous enforcement lapses documented as recently as 2021.^116^

The global trade in plastic waste further complicates the issue. Although amendments to the Basel Convention in 2019 were intended to restrict international transfers of hazardous plastic waste, illegal exports still occur. An increase in illegal shipments from wealthy countries to developing countries, frequently masquerading as recyclables, was noted in a 2020 Interpol report. Exporters including the US, UK, and Germany sent massive amounts of poorly controlled plastic garbage to Southeast Asian nations like Malaysia and Vietnam between 2018 and 2020.^117^ Much of this material ended up in unregulated landfills or incinerators, exacerbating environmental degradation and exposing local populations to harmful substances. Additionally, the lack of harmonized international standards for evaluating the combined risks posed by multiple toxic plastic additives undermines coordinated global action—leaving vulnerable populations at greater risk.^118^

## 4. Innovative approaches to risk reduction and technology

### 4.1. Advanced recycling technologies and their role in sustainable plastic waste management

**Fig. 5:**
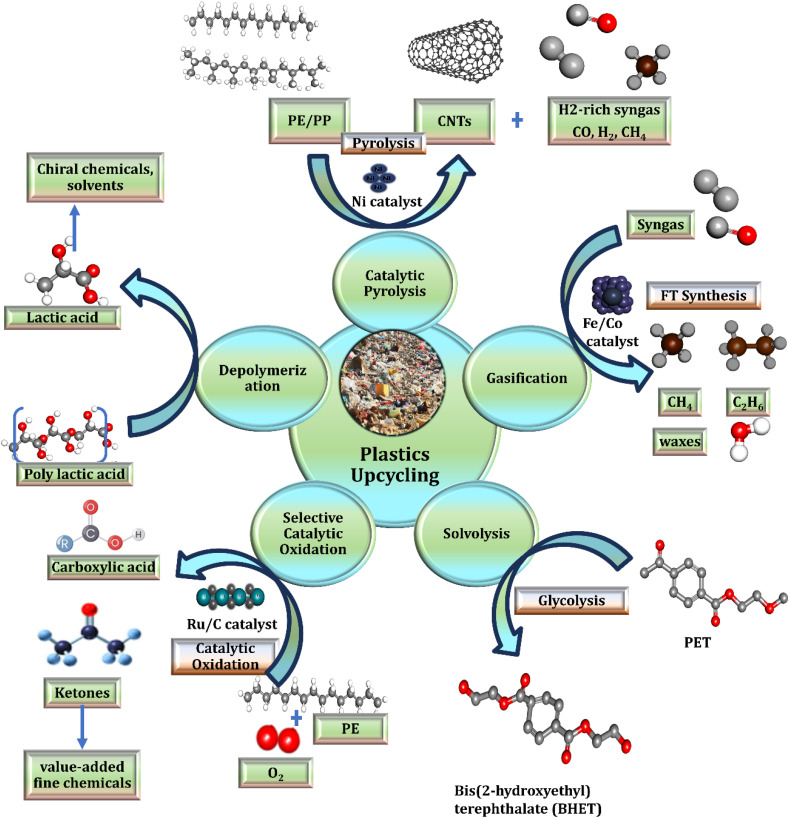
Overview of plastics upcycling pathways and products.

The development of advanced recycling technologies is transforming how the world addresses plastic waste in a more sustainable manner. In contrast to traditional mechanical recycling which often results in lower-quality materials after multiple uses these emerging approaches use chemical and thermal methods to deconstruct plastics at the molecular level. This process enables the creation of high-quality recycled products that are comparable to new, virgin plastics. These technologies are particularly effective in dealing with issues that hinder mechanical recycling, such as material contamination, mixed plastic types, and difficult-to-recycle products like multi-layered packaging. For example, chemical depolymerization has shown promising results in recovering polyethylene terephthalate (PET), achieving recovery rates as high as 97% under ideal conditions.^119^ This schematic illustrates key strategies for transforming plastic waste into value-added chemicals and materials. Major approaches include catalytic pyrolysis (producing carbon nanotubes and H_2_-rich syngas), gasification (yielding syngas and FT-derived fuels/waxes), solvolysis/glycolysis (recovering PET and bis(2-hydroxyethyl) terephthalate, BHET), depolymerization (producing lactic acid and poly(lactic acid)), and selective catalytic oxidation (yielding carboxylic acids and ketones for fine chemicals). Catalysts and reaction conditions are highlighted for each pathway (Fig. 5).

Pyrolysis is a prominent technique that thermally breaks down plastic waste in the absence of oxygen, producing pyrolytic oil. This oil can then be processed into fuels, chemical raw materials, or reused to manufacture new plastics. Countries like the US and Japan are investing more in pyrolysis technology, and businesses like ExxonMobil and Agilyx are using them on a commercial basis. ExxonMobil stated that by 2023, it was turning more than 30 000 tons of plastic per year into oil for the manufacture of new polymers.^120^ Gasification is another important strategy that turns plastic waste into syngas, a mixture of hydrogen in addition to carbon monoxide used in chemical manufacturing and energy production. Germany has integrated gasification into its waste-to-energy facilities, demonstrating how this technology can simultaneously recover energy and recycle waste. Unlike incineration, gasification reduces environmental impacts by capturing and repurposing emissions. Compared with traditional mechanical recycling, pyrolysis and gasification generally require higher energy inputs, but their ability to recover energy and convert waste into valuable products can offset some greenhouse gas emissions, contributing to a potentially lower net carbon footprint if managed correctly. Enzymatic recycling offers additional sustainability benefits, operating under milder conditions with reduced energy demand and lower associated greenhouse gas emissions, making it a promising approach for environmentally friendly plastic waste management. However, the high costs associated with installing and maintaining gasification systems continue to limit adoption in low- and middle-income regions.^121^

A more recent advancement is enzymatic recycling, which uses specially engineered enzymes to depolymerize plastics like PET under relatively mild conditions. This process typically requires lower temperatures and pressures, resulting in reduced energy demand and greenhouse gas emissions. An enzyme created by French researchers can degrade 90% of PET in ten hours at 72 °C. This is a viable method of recycling plastics that are used to package food and drink. In Europe, a number of pilot projects are assessing the economic viability and scalability of enzymatic reprocessing on a bigger scale.^122^ Despite the potential of these technologies, several barriers persist. Most advanced recycling methods require significant financial investment, high energy inputs, and sophisticated infrastructure factors that hinder widespread deployment, particularly in developing countries. Additionally, the environmental trade-offs associated with some methods, such as pyrolysis and gasification, need to be managed carefully to remain aligned with international sustainability goals. However, for lasting waste reduction, it is crucial to implement these technologies within a circular economy model. Nations such as Germany and South Korea have shown that with strong regulatory backing and advanced infrastructure, plastic recycling rates can surpass 50% (OECD, 2024; 2018). These developments not only lessen environmental damage but also promote industrial growth and generate economic benefits in the recycling industry.^123^

### 4.2. Advancements in sustainable bioplastics and eco-friendly polymers

**Fig. 6:**
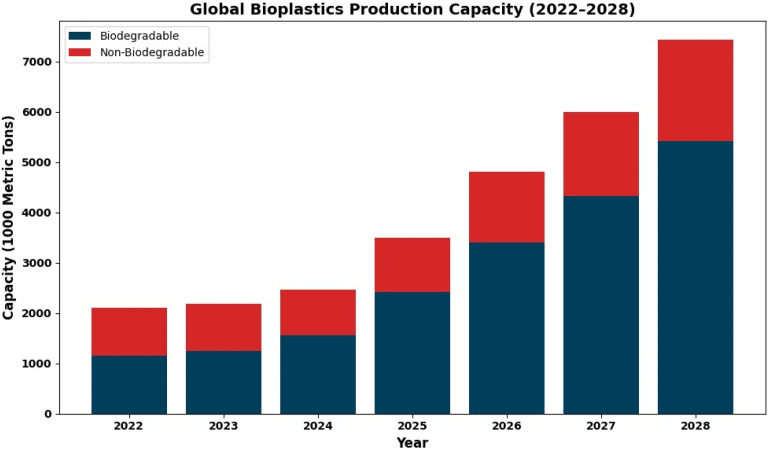
Bioplastics market growth: global production capacity from 2022 to 2028.^124^

A more sustainable substitute for traditional plastics derived from fossil fuels, bioplastics are made from sustainable sources like sugarcane, cellulose, and starch. They aim to lessen reliance on finite resources and typically generate fewer carbon emissions throughout their life cycle. Global production of bioplastics exceeded about 1.8 million metric tons by 2022, out of which about 864 thousand metric tons were biodegradable. As shown in Fig. 6 forecasts indicate a significant rise, with global production projected to climb to nearly 7.4 million metric tons.

This consistent growth is depicted in Fig. 6. The growing use of biodegradable polymers reflects a broader global shift toward sustainable materials, driven by advances in biopolymer technologies and increasingly stringent environmental regulations. Europe has been a major driver of this transition, with countries such as Germany and Italy leading in the adoption of bioplastics across industries including packaging, agriculture, and consumer goods. Common biodegradable polymers include polylactic acid (PLA, (C_3_H_4_O_2_)_*n*_), polyhydroxyalkanoates (PHA, (C_4_H_6_O_2_)_*n*_), and polybutylene succinate (PBS, (C_8_H_14_O_4_)_*n*_), which can be metabolized by microorganisms into biomass, carbon dioxide, and water under appropriate composting conditions. Supportive regulations in these countries actively promote the use of compostable materials, particularly for disposable and food-related applications, helping to reduce plastic pollution and enhance circularity in material management.^125^Table 2 summarizes common biodegradable polymers, their chemical formulas, typical applications, and biodegradation characteristics.

**Table 2:** Common biodegradable polymers: chemical formulas, applications, and biodegradation characteristics^126,127^

Polymer	Chemical formula	Typical applications	Biodegradation characteristics
Polylactic acid (PLA)	(C_3_H_4_O_2_)_*n*_	Packaging, disposable cutlery, fibers	Compostable under industrial conditions; metabolized by microorganisms to CO_2_, water, and biomass
Polyhydroxyalkanoates (PHA)	(C_4_H_6_O_2_)_*n*_	Packaging, agricultural films, medical implants	Biodegradable in soil, freshwater, and marine environments; microbial degradation produces CO_2_, water, and biomass
Polybutylene succinate (PBS)	(C_8_H_14_O_4_)_*n*_	Packaging, disposable products, mulching films	Biodegradable in soil and composting systems; supports microbial growth as a carbon source
Starch-based plastics	(C_6_H_10_O_5_)_*n*_	Bags, packaging, disposable items	Rapidly biodegradable under aerobic conditions; converted to CO_2_, water, and biomass
Polycaprolactone (PCL)	(C_6_H_10_O_2_)_*n*_	Medical devices, specialty films	Biodegradable under composting and soil conditions; enzymatic degradation produces CO_2_ and water

Polylactic acid (PLA) is a commonly used bioplastic derived from renewable agricultural sources such as corn starch and sugarcane. PLA, a type of thermoplastic aliphatic polyester, is widely used in industries such as packaging, 3D printing, and the production of disposable goods because of its strength and transparency.^128^ Unlike petroleum-based plastics, PLA and other bio-based alternatives can partially offset carbon emissions, as the raw materials primarily plants absorb carbon dioxide during their growth. However, this carbon offset varies depending on the material's composition. For example, bio-based polyethylene (bio-PE), with a carbon content of 86%, absorbs around 3.1 kg of CO_2_ per kilogram, while PLA, with 50% carbon content, absorbs about 1.8 kg CO_2_ per kg. Another key category of bioplastics is polyhydroxyalkanoates (PHAs), which are produced using feedstocks derived from plants and microbial fermentation. PHAs are distinct from PLA in that they are completely biodegradable in a variety of conditions, including terrestrial and marine environments. This makes them especially well-suited for uses like medicinal products and agricultural films. Nations like as the United States and Japan are aggressively promoting PHA manufacturing. An example is Danimer Scientific, an American firm that has launched PHA products aimed at addressing ocean plastic pollution.^129^

Beyond bio-based plastics, researchers are also focusing on synthetic materials designed to degrade more rapidly. These include photo-degradable and oxo-degradable plastics, which are engineered to break down under exposure to light oxygen or microbial activity. The real-world environmental performance of certain plastics continues to be contentious. Oxo-degradable plastics, for example, have faced criticism for breaking down into microplastics rather than fully degrading. In response, the European Union prohibited their use in 2021 through the Single-Use Plastics Directive, promoting certified compostable alternatives that align with recognized biodegradability criteria. Meanwhile, interest is growing in fully biodegradable materials like polybutylene succinate (PBS). Nations such as China and South Korea are actively supporting research and production of PBS, signalling a wider shift toward sustainable plastic solutions.^130^

### 4.3. Advances in biodegradable and degradable plastics: synthetic, bio-based, and fungal-assisted decomposition

The increasing interest in bioplastics and degradable polymers reflects their potential to reduce reliance on fossil-based plastics. However, several key challenges hinder their widespread adoption. These include elevated production expenses, limited access to eco-friendly raw materials, and unpredictable degradation performance in varying environmental conditions. While substances such as polylactic acid (PLA) and polyhydroxyalkanoates (PHA) can be efficiently composted under industrial conditions, their breakdown in natural environments like soil and water tends to be much slower.^131^ Moreover, sourcing these materials from food crops raises sustainability concerns, particularly in regions where food security and land availability are already under strain. To address these issues, research efforts are turning toward non-food sources such as crop residues, lignocellulosic biomass, and algae for bioplastic synthesis. Fungi contribute to the decomposition of plastics by adhering to surfaces and penetrating them with hyphae. This process initiates physical breakdown and eventually results in the conversion of the material into biomass, carbon dioxide, and other environmentally benign products. It is anticipated that advancements in biotechnological techniques, bolstered by legislative actions and heightened public involvement, will propel the expansion of environmentally friendly plastic alternatives. Market projections suggest that the bioplastics industry could exceed $45.2 billion in value by 2029, underscoring a shift toward more sustainable material usage and circular economic practices.^132^

### 4.4. Microbial allies in the fight against plastic pollution

Recent breakthroughs in biotechnology have highlighted the potential of using microorganisms to combat plastic pollution by breaking down synthetic polymers and reducing associated environmental toxins. Both bacteria and fungi have shown remarkable capabilities to degrade various types of plastics, providing a biologically sound and sustainable substitute for conventional garbage disposal approaches. This growing area of research investigates how microbes interact with plastic materials, the environmental conditions that enhance their activity, and the scalability of these methods for industrial use, signaling a transformative approach to plastic waste management.^133^

**Fig. 7:**
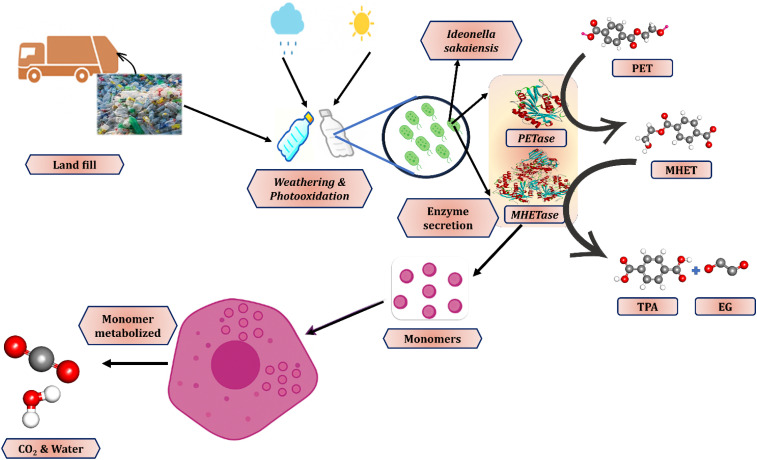
Advanced microbial recycling of plastics. Plastic waste such as PET can be enzymatically degraded by specialized microbes (*e.g.*, *Ideonella sakaiensis*) through the action of PETase and MHETase^134^

Recent research highlights microbial biodegradation as a promising approach for managing polyethylene terephthalate (PET) waste (Fig. 7). In landfills, environmental weathering—including photo-oxidation and mechanical stress—renders plastics more susceptible to microbial action. The bacterium *Ideonella sakaiensis* can adhere to PET surfaces and secrete the enzymes PETase and MHETase, which depolymerize PET into monomers such as terephthalic acid and ethylene glycol. These monomers are then metabolized by the bacteria as carbon and energy sources. This enzymatic process offers an eco-friendly alternative to conventional recycling, operating under mild conditions with lower energy requirements and reduced greenhouse gas emissions. Although challenges remain in terms of degradation rates and scalability, microbial PET degradation represents a complementary strategy for managing end-of-life plastics within a circular economy framework.^135^

**Fig. 8:**
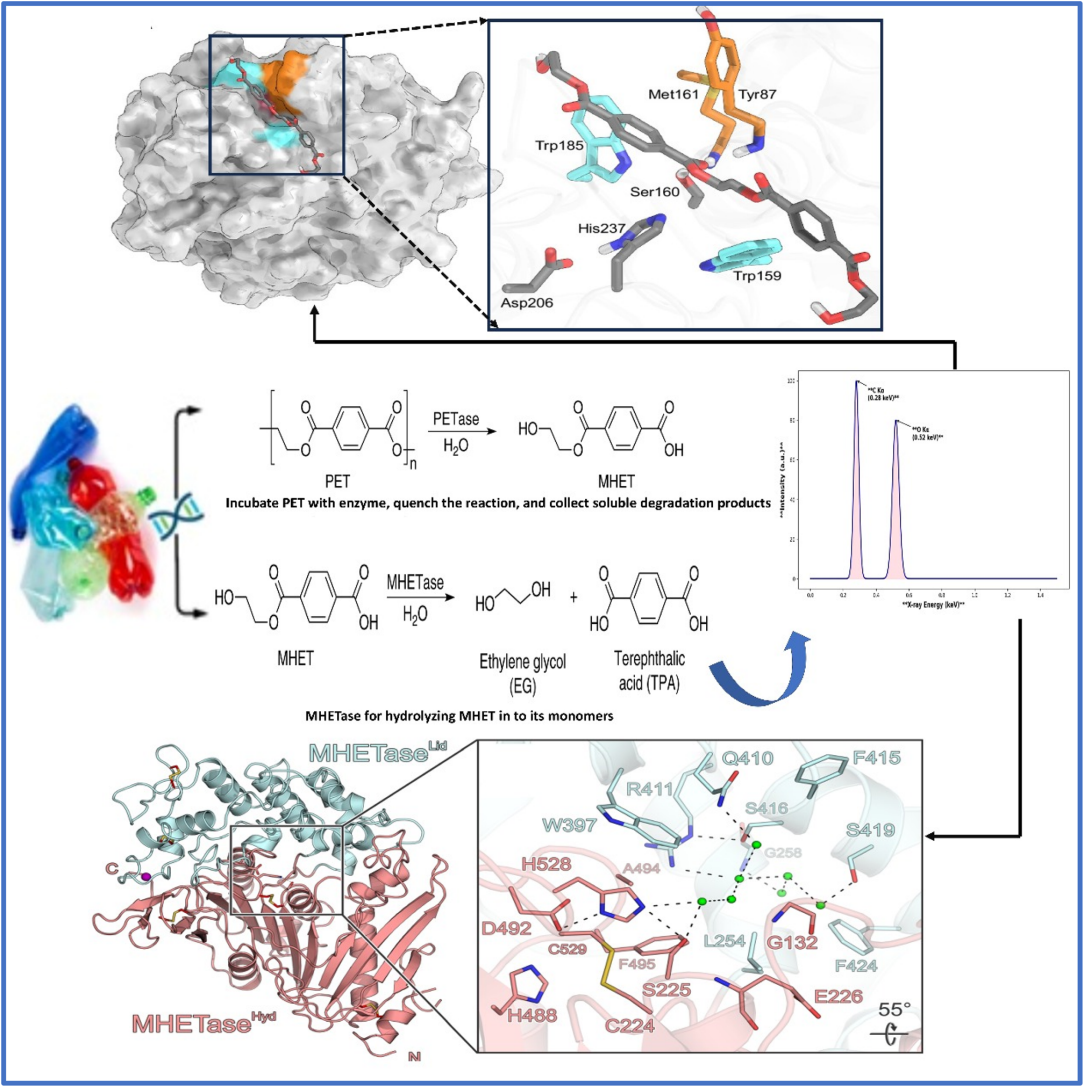
Structural and functional insights into PET degradation by PETase and MHETase enzymes.^138,139^

The microbial breakdown of plastics primarily depends on enzymes that cleave long polymer chains into smaller, more manageable molecules. One of the most well-documented cases involves *Ideonella sakaiensis*, a bacterium known for secreting two enzymes PETase and MHETase that work in tandem to decompose polyethylene terephthalate (PET), commonly used in beverage bottles.^136^ PETase initiates the process by converting PET into mono-(2-hydroxyethyl) terephthalate (MHET), which is then further hydrolyzed by MHETase into terephthalic acid (TPA) and ethylene glycol (EG) both valuable for reuse in plastic manufacturing.^137^ Structural analyses have shown that PETase contains a catalytic triad of serine, histidine, and aspartate, along with a flexible tryptophan residue (Trp185) that enhances its ability to bind to and process the polymer substrate.^114^ Protein engineering techniques, such as directed evolution and modifications for increased thermal resistance, have further improved these enzymes' performance, allowing them to act more effectively on rigid PET materials. Fig. 8 illustrates these biochemical interactions at the molecular level. Comparably, aspergillus niger as well as Penicillium chrysogenum are two fungi that have demonstrated potential in decomposing plastics like polyethylene (PE) along with polystyrene (PS), especially in lab conditions. Studies conducted in China additionally identified soil fungi that, in controlled environments, can break down PE film, which is frequently found in plastic grocery bags, at rates of as high as 20% over a month.^140^

Environmental factors including temperature, oxygen availability, pH, and the existence of extra organic matter all have a significant impact on how well microorganisms break down plastic. According to Brazilian research, marine microorganisms isolated from plastic waste found along the coast have higher rates of disintegration under salty conditions. This implies that microbial remedies can be customized for particular environments, like landfills or oceanic settings.^141^ Simultaneously, research in China has investigated the deployment of genetically modified microorganisms with improved enzymes that demonstrate a greater capacity to degrade durable polymers such as polystyrene (PS) and polyethylene (PE). Notwithstanding these developments, the extent of degradation attained in lab settings still falls well short of the organic materials' natural rate of breakdown, suggesting that more optimization is required.^142^

In order to bring microbial degrading technologies closer to practical use, efforts are being made to integrate them with existing waste treatment systems. Pilot initiatives in countries such as Germany, India, and Spain have tested the introduction of microbial populations into landfill environments a process known as bioaugmentation to improve plastic breakdown. Additionally, specialized bioreactors are being developed to provide controlled conditions that support microbial activity. One project in Ecuador employed a bioreactor featuring *Ideonella sakaiensis*, resulting in a 31% improvement in PET degradation compared to standard methods. These strategies demonstrate the potential of combining biological and chemical techniques to create more comprehensive solutions for plastic waste management.^143^

Despite promising developments, several challenges must be addressed before microbial degradation can be applied on a global scale. The wide variability in plastic composition requires the use of specific microbial strains or enzymes for each type of polymer. Furthermore, deploying genetically modified organisms in open environments raises ecological and regulatory concerns that must be carefully managed.^144^ Future research should prioritize the identification of microbes capable of degrading multiple plastic types and the development of scalable, cost-effective systems suitable for industrial use. By overcoming these obstacles, microbial activities could play a bigger role in combating plastic pollution and help promote more environmentally friendly waste management techniques globally.^145^

## 5. Global strategies and policy solutions for plastic risk reduction

### 5.1. Actionable strategies to address plastic waste impacts

Table 3 highlights the pressing need for stricter laws and better waste management procedures due to the growing worries about the discharge of hazardous compounds from plastic materials. Often present in common plastics, harmful substances like polybrominated diphenyl ethers (PBDEs), phthalates, and BPA can contaminate soil and water. Addressing these threats requires a comprehensive strategy enforcing strict controls on hazardous additives during manufacturing and advancing effective waste treatment systems to reduce environmental contamination.^146^

**Table 3:** Comprehensive overview of plastic waste challenges and solutions

S. No	Key concern	Cause/source	Brief	Risk	Health effects	Mitigation strategy
1	Marine pollution	Littering, river dumping, fishing gear	Plastics enter oceans *via* rivers, coasts, and ships	Harm to marine life, habitat loss, ingestion by aquatic species	Contaminated seafood, potential endocrine disruption *via* microplastics ingestion	Coastal cleanups, waste interception, bans on marine dumping, biodegradable fishing nets
2	Soil and land contamination	Landfills, plastic mulch films, illegal dumping	Plastics leach chemicals into soil, affecting fertility	Soil degradation, impact on crop growth	Uptake of toxicants into food crops, exposure *via* agriculture	Promote compostable alternatives in agriculture, enforce anti-dumping regulations
3	Air pollution	Open burning, incineration without filters	Releases dioxins, furans, and particulate matter	Air contamination, climate change contribution	Respiratory problems, cancers, developmental and reproductive toxicity	Ban open burning, install air filters, use cleaner incineration technology
4	Toxic chemical exposure	Additives like BPA, phthalates, flame retardants	Leaching from plastics during use and disposal	Water and food contamination, bioaccumulation in food chains	Endocrine disruption, developmental effects, cancers	Regulate hazardous additives, switch to safer materials, monitor chemical levels
5	Microplastic pollution	Breakdown of larger plastics, cosmetics, textiles	Tiny plastic particles persist in ecosystems and food chains	Ecosystem toxicity, ingestion by all trophic levels	Unknown long-term risks, potential organ inflammation, immune system disruption	Ban microbeads, improve filtration in wastewater treatment, reduce synthetic clothing use
6	Overuse of single-use plastics	Packaging, cutlery, bags, straws	Convenience leads to excessive plastic production and waste	Waste accumulation, littering, landfill overcapacity	Indirect exposure *via* environment, increase in waste burden	Enforce bans/taxes on single-use plastics, promote reusable alternatives
7	Inadequate waste management	Poor collection, lack of segregation/recycling	Plastics remain in the environment for centuries	Accumulation in natural areas, illegal burning or dumping	Community-wide exposure to toxins, infections from unmanaged waste	Strengthen infrastructure, public-private partnerships, promote source segregation
8	Low public awareness	Lack of education, misinformation	People unaware of disposal practices and health risks	Continued plastic pollution, poor participation in recycling efforts	Increased exposure to pollutants due to mismanagement	Educational campaigns, integrate plastic literacy in schools, media outreach programs

Emissions from open burning and poorly operated incinerators pose serious health threats, releasing airborne toxins like dioxins and furans that can damage respiratory and cardiovascular systems. Shifting toward cleaner technologies, including advanced waste-to-energy solutions, provides a more sustainable method of managing plastic waste while reducing toxic emissions. To ensure safe incineration practices, harmonized regional standards and strict enforcement against unsafe burning are essential. Continuous air quality monitoring near waste processing sites, along with regular health screenings for exposed workers, will further support public health and strengthen oversight.^147^ Landfill contamination from plastic waste presents an additional environmental hazard, threatening soil quality, groundwater safety, and agricultural productivity. Proper landfill design featuring impermeable liners and modern leachate treatment systems is vital to preventing chemical seepage into surrounding ecosystems. Promoting responsible disposal habits and investing in the development of biodegradable alternatives can also reduce long-term environmental damage. Finally, safeguarding the health of workers in recycling and waste management sectors must remain a priority. This includes adequate training, use of protective equipment, and regular health monitoring to mitigate exposure to toxic substances encountered during material handling and processing.^148^

### 5.2. Reducing health risks from plastic food packaging

**Fig. 9:**
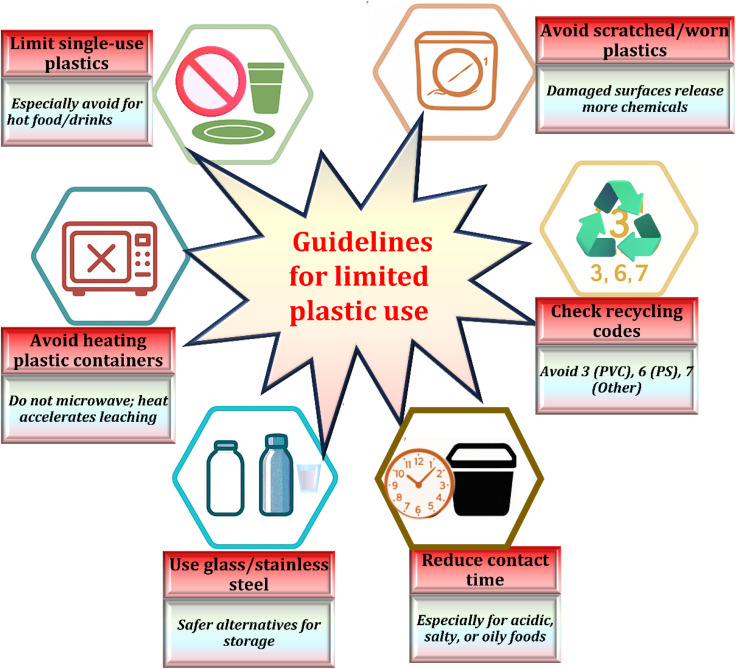
Minimizing toxic additive leaching from plastics into food and beverages: key strategies and safety guidelines for safe plastic use in food contact.^149^

The widespread use of plastic in food packaging and storage has raised increasing concern about its possible hazards to the environment and human health. As shown in Fig. 9, a key safety measure is to avoid heating food in plastic containers. High temperatures can release toxic chemicals such as phthalates and BPA into food and beverages, which have been linked to a number of negative health impacts.^150^ Replacing plastic with safer alternatives like glass or stainless steel offers a healthier and more sustainable approach. Raising public awareness is essential to reducing unsafe practices, such as microwaving food in plastic or using plastic containers for hot or acidic items. Another key measure is limiting the use of single-use plastics, which are major contributors to pollution and are often non-recyclable. Reusable and biodegradable options like bamboo utensils, metal straws, and plant-based dishware offer practical and eco-friendly substitutes that help cut down on plastic waste.^151^

Fig. 9 also underscores the importance of understanding plastic resin codes. The plastics designated with the serial numbers 3 (PVC), 6 (polystyrene), and 7 (other/mixed) are generally not recommended for use in food because they contain potentially hazardous additives. Additionally, it's critical to routinely check containers for wear and tear because discolouration, cracks, and scratches can raise the possibility of chemical leaching. Moreover, minimizing the use of plastic with acidic, oily, or salty foods can further reduce potential health risks. By adopting mindful food storage habits and selecting safer materials, individuals can protect their well-being while contributing to environmental preservation.^152^

### 5.3. Policy measures to tackle plastic waste and its impacts

Addressing the environmental and public health challenges associated with plastic waste calls for a multifaceted policy approach. This involves not only enforcing robust regulations but also promoting the use of sustainable materials and encouraging global cooperation. Across the globe, governments have introduced a range of legal and incentive-driven strategies, from banning select plastic products to mandating corporate accountability and implementing reward-based recycling schemes.^153^ Examining these policy efforts reveals important lessons about their effectiveness, shortcomings, and adaptability across different national and regional settings.^154^

An impactful policy approach is Extended Producer Responsibility (EPR), which places the onus on producers to manage their products throughout their entire lifecycle, including the collection and disposal of waste after consumer use.^155^ Germany's Packaging Act is a notable example, requiring companies to fund the collection and recycling of packaging materials—a move that helped the country achieve a plastic packaging recycling rate exceeding 41% by 2022.^156^ Japan has implemented a similar system through its Containers and Packaging Recycling Law, which compels producers to financially support recycling efforts, contributing to a high recovery rate of over 85%. Nonetheless, these systems still raise concerns over the long-term reliance on energy recovery methods such as incineration, which may undermine broader sustainability goals.^157^

Bans on single-use plastics have also proven effective in curbing plastic waste at its source. Kenya's 2017^158^ ban on plastic bags, which carries strict penalties, resulted in a 90% reduction in usage within a year, leading to noticeable improvements in urban cleanliness and drainage systems. Rwanda has taken a similarly bold stance by outlawing disposable plastics, with its capital Kigali frequently recognized for its clean environment.^159–161^ In Europe, the EU's Single-Use Plastics Directive has limited the availability of items like plastic straws and cutlery, fostering demand for eco-friendly alternatives.^162^ However, these bans often require parallel investments in affordable substitutes, public education targeting schools, communities, and specific industries, and enforcement mechanisms—especially in low-resource contexts—to ensure lasting impact.^163^

Deposit-return systems (DRS) offer another successful example of policy innovation, particularly for managing beverage container waste.^164^ Countries such as Norway and Sweden have implemented highly efficient DRS programs, where consumers receive refunds for returning used containers.^165^ These initiatives, supported by strong coordination among retailers, consumers, and recyclers, have achieved return rates above 95%. However, countries like as Indonesia and India have experimented with smaller scale, community-based DRS projects with promising outcomes, although challenges related to funding, logistics, and long-term scalability remain.^166^

On the international front, policy interventions have sought to create a more just and transparent system for managing plastic waste.^167^ The Basel Convention, especially following its 2021 amendment, now places tighter controls on the cross-border movement of plastic waste, aiming to reduce the export of contaminated or non-recyclable materials to vulnerable regions.^168^ In response, countries like Malaysia have begun rejecting poorly sorted imports and tightening regulatory oversight. In 2024, the United Nations Environment Assembly (UNEA-5.2) approved a resolution to develop a legally binding agreement aimed at addressing plastic pollution.^169^ This initiative highlights the importance of integrating global policy frameworks to support developing nations, ensuring equitable participation in circular economy transitions, and strengthening technical and financial assistance for less-resourced regions.^170^

Despite these advancements, policy effectiveness remains uneven. High-income countries, with their advanced infrastructure and institutional capacity, tend to implement these measures more successfully.^171^ In contrast, developing nations often face significant barriers, including limited financial resources and weak enforcement systems.^172^ The lack of standardized global policies further complicates efforts to address plastic waste as a transboundary issue. Strengthening international cooperation, aligning policy frameworks, and providing targeted support to less-resourced regions are crucial steps toward achieving a more coordinated, inclusive, and effective global response to plastic pollution.^173,174^ By learning from the diverse policy experiences across countries, governments and stakeholders can shape more adaptive, just, and effective strategies to tackle plastic pollution—one of the most urgent environmental and public health threats of our time.^175^

## 6. Conclusion

This review highlights the extensive environmental and health impacts of toxic compounds released from plastic waste, emphasizing primary exposure pathways, associated health risks, and potential mitigation strategies. Plastics persist in ecosystems as sources of hazardous agents, including bisphenol A, phthalates, microplastics, and dioxins, posing disproportionate risks to vulnerable populations, particularly in low-income regions with inadequate regulation and waste management infrastructure. While innovations such as bio-based and biodegradable plastics, photo-degradable and oxo-degradable materials, and advanced chemical recycling technologies offer promising avenues for mitigating these risks, widespread adoption remains constrained by technological challenges, high production costs, and limited resource availability.

Effective management of plastic-related hazards requires a multi-faceted approach. Regulatory tools—including product restrictions, bans on certain single-use plastics, extended producer responsibility (EPR) schemes, and incentives for materials innovation and eco-design—must be integrated with circular economy principles, sustainable product design, and behavioral interventions. Research priorities include developing low-cost, scalable recycling technologies, investigating chronic and cumulative effects of plastic-borne pollutants, and optimizing bio-based and biodegradable alternatives for real-world conditions.

Ultimately, achieving sustainable and equitable plastic management demands cohesive international governance, harmonized standards, and coordinated policies that align technological innovation with environmental and public health objectives. Through such integrated efforts, the transition toward a circular and sustainable plastic economy can be accelerated, reducing the global burden of plastic-associated hazards while promoting environmental resilience.

## Conflicts of interest

There are no conflicts to declare.

## Data Availability

No new data were created or analysed in this study. Data sharing is not applicable to this article.

## References

[cit1] Sharma S., Sharma V., Chatterjee S. (2023). Contribution of plastic and microplastic to global climate change and their conjoining impacts on the environment: a review. Sci. Total Environ..

[cit2] Singh M., Singh M., Singh S. K. (2024). Tackling municipal solid waste crisis in India: insights into cutting-edge technologies and risk assessment. Sci. Total Environ..

[cit3] Singh N., Walker T. R. (2024). Plastic recycling: a panacea or environmental pollution problem. npj Mater. Sustain..

[cit4] Singh S., Malyan S. K., Maithani C., Kashyap S., Tyagi V. K., Singh R. (2023). *et al.*, Microplastics in landfill leachate: occurrence, health concerns, and removal strategies. J. Environ. Manage..

[cit5] Solaja O. M., Osifo O. K., Amoo O. F. (2024). Empowering informal plastic recyclers: addressing socio-economic challenges and human rights awareness in Ogun State, Nigeria. BMC Environ. Sci..

[cit6] Stevens S., McPartland M., Bartosova Z., Skåland H. S., Völker J., Wagner M. (2024). Plastic food packaging from five countries contains endocrine- and metabolism-disrupting chemicals. Environ. Sci. Technol..

[cit7] Swetha T. A., Bora A., Mohanrasu K., Balaji P., Raja R., Ponnuchamy K. (2023). *et al.*, A comprehensive review on polylactic acid (PLA) – synthesis, processing and application in food packaging. Int. J. Biol. Macromol..

[cit8] Synani K., Abeliotis K., Velonia K., Maragkaki A., Manios T., Lasaridi K. (2024). Environmental impact and sustainability of bioplastic production from food waste. Sustainability.

[cit9] Tamizhdurai P., Mangesh V. L., Santhosh S., Vedavalli R., Kavitha C., Bhutto J. K. (2024). *et al.*, A state-of-the-art review of multilayer packaging recycling: challenges, alternatives, and outlook. J. Clean. Prod..

[cit10] Taufiqurrachman S. A. L., Irwandi P. S., Indarti C. F. S. (2024). Public-private partnerships for sustainable urban development: lessons from Indonesian cities. Visioner J. Pemerintah. Drh. Di. Indones..

[cit11] Thomas A. P., Kasa V. P., Dubey B. K., Sen R., Sarmah A. K. (2023). Synthesis and commercialization of bioplastics: organic waste as a sustainable feedstock. Sci. Total Environ..

[cit12] Thushari G. G. N. , Senevirathna J. D. M., Wijesena N. M. and De Zoysa H. K. S., Management of marine plastic debris and microplastics. in Waste Technology for Emerging Economies, Boca Raton, CRC Press, 2022, p. 79–109, 10.1201/9781003132349-5

[cit13] Times of India , India Produces Most Plastic Pollution in the World, Contributes 20% of Global Waste: a Study Finds, The Times of India, 2024, cited 2025 Jan 13, available from: https://timesofindia.indiatimes.com/world/india-produces-most-plastic-pollution-in-the-world-contributes-20-of-global-waste-a-study-finds/articleshow/113257562.cms#

[cit14] Tournier V., Topham C. M., Gilles A., David B., Folgoas C., Moya-Leclair E. (2020). *et al.*, An engineered PET depolymerase to break down and recycle plastic bottles. Nature.

[cit15] Tumwesigye E., Felicitas Nnadozie C., Akamagwuna C. F., Siwe Noundou X., William Nyakairu G., Odume O. N. (2023). Microplastics as vectors of chemical contaminants and biological agents in freshwater ecosystems: current knowledge status and future perspectives. Environ. Pollut..

[cit16] Turner A., Filella M. (2021). Hazardous metal additives in plastics and their environmental impacts. Environ.
Int..

[cit17] Ullah S., Ahmad S., Guo X., Ullah S., Ullah S., Nabi G. (2023). *et al.*, A review of the endocrine disrupting effects of micro and nano plastic and their associated chemicals in mammals. Front. Endocrinol..

[cit18] Ullah Z., Peng L., Lodhi A. F., Kakar M. U., Mehboob M. Z., Iqbal I. (2024). The threat of microplastics and microbial degradation potential: a current perspective. Sci. Total Environ..

[cit19] Massahi T., Omer A. K., Kiani A., Mansouri B., Soleimani H., Fattahi N. (2025). *et al.*, A simulation study on the temperature-dependent release of endocrine-disrupting chemicals from polypropylene and polystyrene containers. Sci. Rep..

[cit20] Seref N., Cufaoglu G. (2025). Food packaging and chemical migration: a food safety perspective. J. Food Sci..

[cit21] Gonsioroski A., Mourikes V. E., Flaws J. A. (2020). Endocrine disruptors in water and their effects on the reproductive system. Int. J. Mol. Sci..

[cit22] Adam I., Walker T. R., Bezerra J. C., Clayton A. (2020). Policies to reduce single-use plastic marine pollution in West Africa. Mar. Policy.

[cit23] Adedara M. L., Taiwo R., Bork H.-R. (2023). Municipal solid waste collection and coverage rates in sub-Saharan African countries: a comprehensive systematic review and meta-analysis. Waste.

[cit24] Adeoye A. O. , Yelwa J. M., Imam N., Quadri R. O., Lawal O. S., Malomo D. and *et al.*, Pyrolysis of plastic wastes towards achieving a circular economy: an advanced chemistry and technical approach, 2024, p. 215 259, 10.1007/978-981-97-0437-8_11

[cit25] Ait-Touchente Z., Khellaf M., Raffin G., Lebaz N., Elaissari A. (2024). Recent advances in polyvinyl chloride (PVC) recycling. Polym. Adv. Technol..

[cit26] Aldeli N., Murphy D., Hanano A. (2024). Impact of dioxins on reproductive health in female mammals. Front. Toxicol..

[cit27] Alhaj Hamoud Y., Shaghaleh H., Zia-ur-Rehman M., Rizwan M., Umair M., Usman M. (2024). *et al.*, Cadmium and lead accumulation in important food crops due to wastewater irrigation: pollution index and health risks assessment. Heliyon.

[cit28] Ali N., Katsouli J., Marczylo E. L., Gant T. W., Wright S., Bernardino de la Serna J. (2024). The potential impacts of micro- and nano-plastics on various organ systems in humans. EBioMedicine.

[cit29] Almeida-Toledano L., Navarro-Tapia E., Sebastiani G., Ferrero-Martínez S., Ferrer-Aguilar P., García-Algar O. (2024). *et al.*, Effect of prenatal phthalate exposure on fetal development and maternal/neonatal health consequences: a systematic review. Sci. Total Environ..

[cit30] Alvarez-Barragán J., Domínguez-Malfavón L., Vargas-Suárez M., González-Hernández R., Aguilar-Osorio G., Loza-Tavera H. (2016). Biodegradative activities of selected environmental fungi on a polyester polyurethane varnish and polyether polyurethane foams. Appl. Environ. Microbiol..

[cit31] Amobonye A., Bhagwat P., Singh S., Pillai S. (2021). Plastic biodegradation: frontline microbes and their enzymes. Sci. Total Environ..

[cit32] Ankit S., Kumar V., Tiwari J., Sweta R. S., Singh J., Bauddh K. (2021). Electronic waste and their leachates impact on human health and environment: global ecological threat and management. Environ. Technol. Innov..

[cit33] Apriadi B. F., Setiawan R. P., Firmansyah I. (2024). Policy scenario of plastic waste mitigation in Indonesia using system dynamics. Waste Manag. Res..

[cit34] Sheriff S. S., Yusuf A. A., Akiyode O. O., Hallie E. F., Odoma S., Yambasu R. A. (2025). *et al.*, A comprehensive review on exposure to toxins and health risks from plastic waste: challenges, mitigation measures, and policy interventions. Waste Manag. Bull..

[cit35] Baca D., Monroy R., Castillo M., Elkhazraji A., Farooq A., Ahmad R. (2023). Dioxins and plastic waste: a scientometric analysis and systematic literature review of the detection methods. Environ. Adv..

[cit36] Baptista Neto J. A., Gaylarde C., Beech I., Bastos A. C., da Silva Quaresma V., de Carvalho D. G. (2019). Microplastics and attached microorganisms in sediments of the Vitória bay estuarine system in SE Brazil. Ocean Coast Manag..

[cit37] Barboza L. G. A., Cunha S. C., Monteiro C., Fernandes J. O., Guilhermino L. (2020). Bisphenol A and its analogs in muscle and liver of fish from the North East Atlantic Ocean in relation to microplastic contamination: exposure and risk to human consumers. J. Hazard. Mater..

[cit38] Basak S., Das M. K., Duttaroy A. K. (2020). Plastics-derived endocrine-disrupting compounds and their effects on early development. Birth Defects Res. Res.

[cit39] Behuria P. (2021). Ban the (plastic) bag? Explaining variation in the implementation of plastic bag bans in Rwanda, Kenya and Uganda. Environ. Plan. C Politics Space.

[cit40] Miller M. E., Hamann M., Kroon F. J. (2020). Bioaccumulation and biomagnification of microplastics in marine organisms: a review and meta-analysis of current data. PLoS One.

[cit41] Chen S. and Redkar-Palepu V., Umuganda: Rwanda's Audacity of Hope to End Plastic Pollution, United Nations Development Programme (UNDP) Blog, 2023, available from: https://www.undp.org/blog/umuganda-rwandas-audacity-hope-end-plastic-pollution

[cit42] Cheng Y., Yang Y., Bai L., Cui J. (2024). Microplastics: an often-overlooked issue in the transition from chronic inflammation to cancer. J. Transl. Med..

[cit43] World Health Organization , Chapter 4: Solid waste. in Compendium of WHO and other UN guidance on health and environment, 2024 update, Geneva, World Health Organization, 2024, available from: https://iris.who.int/handle/10665/378095

[cit44] Braun J. M. (2017). Early-life exposure to EDCs: role in childhood obesity and neurodevelopment. Nat. Rev. Endocrinol..

[cit45] Briffa J., Sinagra E., Blundell R. (2020). Heavy metal pollution in the environment and their toxicological effects on humans. Heliyon.

[cit46] Burgin T., Pollard B. C., Knott B. C., Mayes H. B., Crowley M. F., McGeehan J. E. (2024). *et al.*, The reaction mechanism of the *Ideonella sakaiensis* PETase enzyme. Commun. Chem..

[cit47] Das S., Sultana K. W., Ndhlala A. R., Mondal M., Chandra I. (2023). Heavy metal pollution in the environment and its impact on health: exploring green technology for remediation. Environ. Health Insights.

[cit48] Ciuffi B., Fratini E., Rosi L. (2024). Plastic pretreatment: the key for efficient enzymatic and biodegradation processes. Polym. Degrad. Stab..

[cit49] Collin S., Baskar A., Geevarghese D. M., Ali M. N. V., Bahubali P., Choudhary R. (2022). *et al.*, Bioaccumulation of lead (Pb) and its effects in plants: a review. J. Hazard. Mater. Lett..

[cit50] Biermann L., Brepohl E., Eichert C., Paschetag M., Watts M., Scholl S. (2021). Development of a continuous PET depolymerization process as a basis for a back-to-monomer recycling method. Green Process. Synth..

[cit51] (a) BMUV , Waste management in Germany 2023: facts, data, figures, Federal Ministry for the Environment, Nature Conservation, Nuclear Safety and Consumer Protection, Berlin, 2023

[cit52] De Paula L. C. P., Alves C. (2024). Food packaging and endocrine disruptors. J. Pediatr..

[cit53] Dehghani S., Moore F., Akhbarizadeh R. (2017). Microplastic pollution in deposited urban dust, Tehran metropolis, Iran. Environ. Sci. Pollut. Res..

[cit54] Shibamoto T., Yasuhara A., Katami T. (2007). Dioxin formation from waste incineration. Rev. Environ. Contam. Toxicol..

[cit55] Lenoir D., Kaune A., Hutzinger O., Mutzenich H., Horch K. (1986). Mechanism of formation of polychlorinated dibenzo-p-dioxins and dibenzofurans (PCDD/Fs). Environ. Sci. Technol..

[cit56] Dey S., Rajak P., Sen K. (2024). Bioaccumulation of metals and metalloids in seafood: a comprehensive overview of mobilization, interactive effects in eutrophic environments, and implications for public health risks. J. Trace Elem. Miner..

[cit57] Dhali S. L., Parida D., Kumar B., Bala K. (2024). Recent trends in microbial and enzymatic plastic degradation: a solution for plastic pollution predicaments. Biotechnol. Sustain. Mater..

[cit58] Turner A., Filella M. (2021). Hazardous metal additives in plastics and their environmental impacts. Environ. Int..

[cit59] Du C., Li Z. (2023). Contamination and health risks of heavy metals in the soil of a historical landfill in northern China. Chemosphere.

[cit60] Dueñas-Moreno J., Mora A., Cervantes-Avilés P., Mahlknecht J. (2022). Groundwater contamination pathways of phthalates and bisphenol A: origin, characteristics, transport, and fate – a review. Environ. Int..

[cit61] World Health Organization , Childhood lead poisoning, WHO, Geneva, 2010, available from: https://www.who.int/news-room/fact-sheets/detail/lead-poisoning-and-health

[cit62] U.S. Environmental Protection Agency (EPA) , Lead in soil, 2023, available from: https://www.epa.gov/lead

[cit63] Pure Earth , Transforming Agbogbloshie from toxic e-waste dump into model recycling center, 2015, available from: https://www.pureearth.org/photos-transforming-agbogbloshie-from-toxic-e-waste-dump-into-model-recycling-center/

[cit64] Dueñas-Moreno J., Mora A., Kumar M., Meng X. Z., Mahlknecht J. (2023). Worldwide risk assessment of phthalates and bisphenol A in humans: the need for updating guidelines. Environ. Int..

[cit65] Dueñas-Moreno J., Vázquez-Tapia I., Mora A., Cervantes-Avilés P., Mahlknecht J., Capparelli M. V. (2024). *et al.*, Occurrence, ecological and health risk assessment of phthalates in a polluted urban river used for agricultural land irrigation in central Mexico. Environ. Res..

[cit66] Edo G. I., Samuel P. O., Oloni G. O., Ezekiel G. O., Ikpekoro V. O., Obasohan P. (2024). *et al.*, Environmental persistence, bioaccumulation, and ecotoxicology of heavy metals. Chem. Ecol..

[cit67] Hann S. , Ettlinger S., Gibbs A. and Hogg D., The Impact of the Use of “Oxo-degradable” Plastic on the Environment, European Commission, 2016, 10.2779/992559

[cit68] Haq F., Kiran M., Khan I. A., Mehmood S., Aziz T., Haroon M. (2025). Exploring the pathways to sustainability: a comprehensive review of biodegradable plastics in the circular economy. Mater. Today Sustain..

[cit69] Hasan M. M., Tarannum M. N. (2025). Adverse impacts of microplastics on soil physicochemical properties and crop health in agricultural systems. J. Hazard. Mater. Adv..

[cit70] European Food Safety Authority (EFSA) , A Coordinated Approach to Assess the Human Health Risks of Micro- and Nanoplastics in Food: Proceedings/Scientific Colloquium (Scientific Colloquium 25), Parma: EFSA, 2021

[cit71] World Health Organization (WHO) , Dietary and Inhalation Exposure to Nano- and Microplastic Particles and Potential Implications for Human Health, Geneva, WHO, 2022

[cit72] Feng Y., Tu C., Li R., Wu D., Yang J., Xia Y. (2023). *et al.*, A systematic review of the impacts of exposure to micro- and nano-plastics on human tissue accumulation and health. Eco-Environ. Health.

[cit73] Ramsperger A. F. R. M., Bergamaschi E., Panizzolo M., Fenoglio I., Laforsch C. (2023). Nano- and microplastics: a comprehensive review on their exposure routes, translocation, and fate in humans. NanoImpact.

[cit74] Alqahtani S., Alqahtani S., Saquib Q., Mohiddin F. (2023). Toxicological impact of microplastics and nanoplastics on humans: understanding the mechanistic aspect of the interaction. Front. Toxicol..

[cit75] European Food Safety Authority (EFSA), Gergelová P., Martino L., Rovesti E. (2025). EFSA scientific report on dietary exposure to lead in the European population. EFSA J..

[cit76] Shahsavani A., Pasalari H., Kermani M., Tangestani M., Ahmadi F. (2025). Health outcomes attributed to inhalation of microplastic released from masks during the COVID-19 pandemic: a systematic review. J. Hazard. Mater. Adv..

[cit77] Hassan S., Thacharodi A., Priya A., Meenatchi R., Hegde T. A., Nguyen H. (2024). *et al.*, Endocrine disruptors: unravelling the link between chemical exposure and women's reproductive health. Environ. Res..

[cit78] He L., Gielen G., Bolan N. S., Zhang X., Qin H., Huang H. (2015). *et al.*, Contamination and remediation of phthalic acid esters in agricultural soils in China: a review. Agron. Sustain. Dev..

[cit79] Huang J., Li X., Zeng G., Cheng X., Tong H., Wang D. (2018). Thermal decomposition mechanisms of poly(vinyl chloride): a computational study. Waste Manag..

[cit80] Mahmud F., Sarker D. B., Jocelyn J. A., Sang Q. X. A. (2024). Molecular and cellular effects of microplastics and nanoplastics: focus on inflammation and senescence. Cells.

[cit81] Huang S., Wang H., Ahmad W., Ahmad A., Ivanovich Vatin N., Mohamed A. M., Deifalla A. F., Mehmood I. (2022). Plastic waste management strategies and their environmental aspects: a scientometric analysis and comprehensive review. Int. Res. J. Publ. Environ. Health.

[cit82] Huang Y. Q., Wong C. K. C., Zheng J. S., Bouwman H., Barra R., Wahlström B., Neretin L., Wong M. H. (2012). Bisphenol A (BPA) in China: a review of sources, environmental levels, and potential human health impacts. Environ. Int..

[cit83] Islam M., Xayachak T., Haque N., Lau D., Bhuiyan M., Pramanik B. K. (2024). Impact of bioplastics on environment from its production to end-of-life. Process Saf. Environ. Prot..

[cit84] Jahandari A. (2023). Microplastics in the urban atmosphere: sources, occurrences, distribution, and potential health implications. J. Hazard. Mater. Adv..

[cit85] Jambeck J. R., Geyer R., Wilcox C., Siegler T. R., Perryman M., Andrady A., Narayan R., Law K. L. (2015). Plastic waste inputs from land into the ocean. Science.

[cit86] Jayasinghe R. R., Abeyrathna W. P., Jayasingha K. R., Hendawitharana M. P., Bandara T. S., Liyanage C. L., Williams K. S. (2023). Exploring the plastic collection and recycling trends in Sri Lanka. Recycling.

[cit87] Lim X. (2021). Microplastics are everywhere—but are they harmful?. Nature.

[cit88] Lane T., Wardani I., Koelmans A. A. (2025). Exposure scenarios for human health risk assessment of nano- and microplastic particles. Microplast. Nanoplast..

[cit89] Jiang Y., Song G., Zhang H. (2023). Material identification and heavy metal characteristics of plastic packaging bags used in Chinese express delivery. Front. Environ. Sci..

[cit90] Jung H., Shin G., Kwak H., Hao L. T., Jegal J., Kim H. J., Jeon H., Park J., Oh D. X. (2023). Review of polymer technologies for improving the recycling and upcycling efficiency of plastic waste. Chemosphere.

[cit91] Kahn L. G., Han X., Koshy T. T., Shao Y., Chu D. B., Kannan K., Trasande L. (2018). Adolescents exposed to the World Trade Center collapse have elevated serum dioxin and furan concentrations more than 12 years later. Environ. Int..

[cit92] World Health Organization , Chapter 4: Solid waste, in Compendium of WHO and other UN guidance on health and environment, 2024 update, Geneva, World Health Organization, 2024, https://iris.who.int/handle/10665/3780951

[cit93] Kenya Plastics Pact. Kenya Plastics Pact & WWF-Kenya drive plastic recycling efforts amid EPR implementation, 2024, https://kpp.or.ke/2024/03/08/kenya-plastics-pact-wwf-kenya-drive-plastic-recycling-efforts-amid-epr-implementation

[cit94] Almack A. , India emerges as the world's largest plastic polluter: what went wrong and what's next?, Plastics for Change, 2024, https://www.plasticsforchange.org/blog/india-emerges-as-the-worlds-largest-plastic-polluter-what-went-wrong-and-whats-next

[cit95] Khan I., Chowdhury S., Techato K. (2022). Waste to energy in developing countries—a rapid review: opportunities, challenges, and policies in selected countries of Sub-Saharan Africa and South Asia towards sustainability. Sustainability.

[cit96] Organisation for Economic Co-operation and Development (OECD) , Waste Management and the Circular Economy in Selected OECD Countries: Evidence from Environmental Performance Reviews, Paris, OECD Publishing, 2019, 10.1787/9789264309395-en

[cit97] Organisation for Economic Co-operation and Development (OECD) , Financing a future free from plastic leakage, Paris, OECD Publishing, 2023, https://www.oecd.org/content/dam/oecd/en/publications/reports/2023/01/financing-a-future-free-from-plastic-leakage_5adb3700/32424f03-en.pdf

[cit98] Kamaludin R., Rasdi Z., Othman M. H. D., Kadir S. A. H. (2024). Human metabolic effects of BPA and the application of a hybrid photocatalytic membrane for BPA-contaminated water. Sustain. Environ. Res..

[cit99] Kanan M., Ramadan M., Haif H., Abdullah B., Mubarak J., Ahmad W., Mari S., Hassan S., Eid R., Hasan M., Qahl M., Assiri A., Sultan M., Alrumaih F., Alenzi A. (2023). Empowering low- and middle-income countries to combat AMR by minimal use of antibiotics: a way forward. Antibiotics.

[cit100] Kanan S., Samara F. (2018). Dioxins and furans: a review from chemical and environmental perspectives. Trends Environ. Anal. Chem..

[cit101] Kassab A., Al Nabhani D., Mohanty P., Pannier C., Ayoub G. Y. (2023). Advancing plastic recycling: challenges and opportunities in the integration of 3D printing and distributed recycling for a circular economy. Polymers.

[cit102] Lestari P., Purwiandono G., Amalia A. N., Ma'Rufi E. K. I., Firdaus M. R., Wacano D. (2025). Coexistence of microplastic particles and heavy metals in landfill leachate: a case study of a landfill in Indonesia. Case Stud. Chem. Environ. Eng..

[cit103] Li K., Ward H., Lin H. X., Tukker A. (2024). Economic viability requires higher recycling rates for imported plastic waste than expected. Nat. Commun..

[cit104] Liang J., Li C., Dang Y., Feng X., Ji X., Liu X., Zhao X., Zhang Q., Ren Z., Wang Y., Li Y., Qu G., Liu R. (2024). Occurrence of bisphenol A analogues in the aquatic environment and their behaviours and toxicity effects in plants. Environ. Int..

[cit105] Liu W., Liao H., Wei M., Junaid M., Chen G., Wang J. (2024). Biological uptake, distribution and toxicity of micro(nano)plastics in the aquatic biota: a special emphasis on size-dependent impacts. TrAC, Trends Anal. Chem..

[cit106] Liu Y., Zhu R., Xu T., Chen Y., Ding Y., Zuo S., Xu L., Xie H. Q., Zhao B. (2024). Potential AhR-independent mechanisms of 2,3,7,8-tetrachlorodibenzo-p-dioxin inhibition of human glioblastoma A172 cells migration. Ecotoxicol. Environ. Saf..

[cit107] Lokesh P., Shobika R., Omer S., Reddy M., Saravanan P., Rajeshkannan R., Saravanan V., Venkatkumar S. (2023). Bioremediation of plastics by the help of microbial tool: a way for control of plastic pollution. Sustain. Chem. Environ..

[cit108] Luís C., Algarra M., Câmara J., Perestrelo R. (2021). Comprehensive insight from phthalates occurrence: from health outcomes to emerging analytical approaches. Toxics.

[cit109] Ma W., Huang Z., Cui J., Boré A., Chen G., Qiao Z., Lou Z., Fellner J. (2023). Inhalation health risk assessment of incineration and landfill in the Bohai Rim, China. Chemosphere.

[cit110] Krivohlavek A., Mikulec N., Budec M., Barusic L., Bosnir J., Sikic S., Jakasa I., Begovic T., Janda R., Vitale K. (2023). Migration of BPA from food packaging and household products on the Croatian market. Int. Res. J. Publ. Environ. Health.

[cit111] Kudzin M. H., Piwowarska D., Festinger N., Chrusciel J. J. (2023). Risks associated with the presence of polyvinyl chloride in the environment and methods for its disposal and utilization. Materials.

[cit112] Kumari T. and Raghubanshi A. S., Waste management practices in the developing nations: challenges and opportunities. in Waste Management and Resource Recycling in the Developing World, Elsevier, 2023, p. 773–797, 10.1016/B978-0-323-90463-6.00017-8

[cit113] Kusuma H. S., Sabita A., Putri N. A., Azliza N., Illiyanasafa N., Darmokoesoemo H. (2024). *et al.*, Waste to wealth: Polyhydroxyalkanoates (PHA) production from food waste for a sustainable packaging paradigm. Food Chem.: Mol. Sci..

[cit114] Lahimer M., Abou Diwan M., Montjean D., Cabry R., Bach V., Ajina M. (2023). *et al.*, Endocrine disrupting chemicals and male fertility: from physiological to molecular effects. Front. Public Health.

[cit115] Lai H., Liu X., Qu M. (2022). Nanoplastics and human health: hazard identification and biointerface. Nanomaterials.

[cit116] Larrain M., Billen P., Van Passel S. (2022). The effect of plastic packaging recycling policy interventions as a complement to extended producer responsibility schemes: a partial equilibrium model. Waste Manag..

[cit117] Lebbie T. S., Moyebi O. D., Asante K. A., Fobil J., Brune-Drisse M. N., Suk W. A. (2021). *et al.*, E-waste in Africa: a serious threat to the health of children. Int. Res. J. Publ. Environ. Health.

[cit118] Lee Y., Cho J., Sohn J., Kim C. (2023). Health effects of microplastic exposures: current issues and perspectives in South Korea. Yonsei Med. J..

[cit119] Moghimi Dehkordi M., Pournuroz Nodeh Z., Soleimani Dehkordi K., Salmanvandi H., Rasouli Khorjestan R., Ghaffarzadeh M. (2024). Soil, air, and water pollution from mining and industrial activities: sources of pollution, environmental impacts, and prevention and control methods. Results Eng..

[cit120] Mohan N., Usha R. (2018). Bio-augmentation – effective method of treating plastic waste – a field study. J. Pure Appl. Microbiol..

[cit121] Motlagh Z. K., Tavakoli M., Sayadi M. H. (2025). Microplastics and heavy metals in the coastal areas: marine health assessment and ecosystem services values. Environ. Dev..

[cit122] Mukheed M., Khan A. (2020). Plastic pollution in Pakistan: environmental and health implications. J. Pollut. Eff. Cont..

[cit123] Pilapitiya N. T. P. G., Ratnayake A. S. (2024). The world of plastic waste: a review. Clean Mater..

[cit124] Nene A., Sadeghzade S., Viaroli S., Yang W., Uchenna U. P., Kandwal A. (2025). *et al.*, Recent advances and future technologies in nano-microplastics detection. Environ. Sci. Eur..

[cit125] Ng C. H., Mistoh M. A., Teo S. H., Galassi A., Ibrahim A., Sipaut C. S. (2023). *et al.*, Plastic waste and microplastic issues in Southeast Asia. Front. Environ. Sci..

[cit126] Boey J. Y., Mohamad L., Khok Y. S., Tay G. S., Baidurah S. (2021). A review of the applications and biodegradation of polyhydroxyalkanoates and poly(lactic acid) and its composites. Polymers.

[cit127] Jha S., Akula B., Enyioma H., Novak M., Amin V., Liang H. (2024). Biodegradable biobased polymers: a review of the state of the art, challenges, and future directions. Polymers.

[cit128] Nguyen A. T., Nguyen N., Phung P., Yen-Khanh N. (2023). Residents' waste management practices in a developing country: a social practice theory analysis. Environ. Challenges.

[cit129] Zhou W., Bergsma S., Colpa D. I., Euverink G. J. W., Krooneman J. (2023). Polyhydroxyalkanoates (PHAs) synthesis and degradation by microbes and applications towards a circular economy. J. Environ. Manage..

[cit130] Ziani K., Ionita-Mindrican C. B., Mititelu M., Neacsu S. M., Negrei C., Morosan E. (2023). *et al.*, Microplastics: a real global threat for environment and food safety: a state of the art review. Nutrients.

[cit131] Zimmermann L., Bartosova Z., Braun K., Oehlmann J., Völker C., Wagner M. (2021). Plastic products leach chemicals that induce *in vitro* toxicity under realistic use conditions. Environ. Sci. Technol..

[cit132] Yuan M., Chen S., Zeng C., Fan Y., Ge W., Chen W. (2023). Estrogenic and non-estrogenic effects of bisphenol A and its action mechanism in the zebrafish model: an overview of the past two decades of work. Environ. Int..

[cit133] Yukioka S., Tanaka S., Nabetani Y., Suzuki Y., Ushijima T., Fujii S. (2020). *et al.*, Occurrence and characteristics of microplastics in surface road dust in Kusatsu (Japan), Da Nang (Vietnam), and Kathmandu (Nepal). Environ. Pollut..

[cit134] Vaishnav A. , Lal J., Singh N. S., Pati B. K., Mehta N. K. and Priyadarshini M. B., Role of microbial enzymes and their modification for plastic biodegradation, in Advanced Strategies for Biodegradation of Plastic Polymers, ed. Soni R., Debbarma P., Suyal D. C. and Goel R., Cham, Springer, 2024, p. 1–16, 10.1007/978-3-031-55661-6_16

[cit135] Yoshida S. (2016). *et al.*, A bacterium that degrades and assimilates poly(ethylene terephthalate). Science.

[cit136] Yusuf A. A., Ampah J. D., Veza I., Atabani A. E., Hoang A. T., Nippae A. (2023). *et al.*, Investigating the influence of plastic waste oils and acetone blends on diesel engine combustion, pollutants, morphological and size particles: dehalogenation and catalytic pyrolysis of plastic waste. Energy Convers. Manag..

[cit137] Yusuf A. A., Dankwa Ampah J., Soudagar M. E. M., Veza I., Kingsley U., Afrane S. (2022). *et al.*, Effects of hybrid nanoparticle additives in n-butanol/waste plastic oil/diesel blends on combustion, particulate and gaseous emissions from diesel engine evaluated with entropy-weighted PROMETHEE II and TOPSIS: environmental and health risks of plastic waste. Energy Convers. Manag..

[cit138] Turner A., Filella M. (2021). Hazardous metal additives in plastics and their environmental impacts. Environ. Int..

[cit139] Ullah S., Ahmad S., Guo X., Ullah S., Ullah S., Nabi G. (2023). *et al.*, A review of the endocrine disrupting effects of micro and nano plastic and their associated chemicals in mammals. Front. Endocrinol..

[cit140] Ullah Z., Peng L., Lodhi A. F., Kakar M. U., Mehboob M. Z., Iqbal I. (2024). The threat of microplastics and microbial degradation potential; a current perspective. Sci. Total Environ..

[cit141] Zhang Y., Pedersen J. N., Eser B. E., Guo Z. (2022). Biodegradation of polyethylene and polystyrene: from microbial deterioration to enzyme discovery. Biotechnol. Adv..

[cit142] Zhao B., Rehati P., Yang Z., Cai Z., Guo C., Li Y. (2024). The potential toxicity of microplastics on human health. Sci. Total Environ..

[cit143] Zhao H., Federigi I., Verani M., Carducci A. (2023). Organic pollutants associated with plastic debris in marine environment: a systematic review of analytical methods, occurrence, and characteristics. Int. Res. J. Publ. Environ. Health.

[cit144] Thomas A. P., Kasa V. P., Dubey B. K., Sen R., Sarmah A. K. (2023). Synthesis and commercialization of bioplastics: organic waste as a sustainable feedstock. Sci. Total Environ..

[cit145] Thushari G. G. N. , Senevirathna J. D. M., Wijesena N. M. and De Zoysa H. K. S., Management of marine plastic debris and microplastics. in Waste Technology for Emerging Economies, Boca Raton, CRC Press, 2022, pp. 79–109, 10.1201/9781003132349-5

[cit146] Tournier V., Topham C. M., Gilles A., David B., Folgoas C., Moya-Leclair E. (2020). *et al.*, An engineered PET depolymerase to break down and recycle plastic bottles. Nature.

[cit147] Tumwesigye E., Nnadozie C. F., Akamagwuna C. F., Siwe Noundou X., Nyakairu G. W., Odume O. N. (2023). Microplastics as vectors of chemical contaminants and biological agents in freshwater ecosystems: current knowledge status and future perspectives. Environ. Pollut..

[cit148] Turner A., Filella M. (2021). Hazardous metal additives in plastics and their environmental impacts. Environ. Int..

[cit149] Viel T., Manfra L., Zupo V., Libralato G., Cocca M., Costantini M. (2023). Biodegradation of plastics induced by marine organisms: future perspectives for bioremediation approaches. Polymers.

[cit150] Ullah S., Ahmad S., Guo X., Ullah S., Ullah S., Nabi G. (2023). *et al.*, A review of the endocrine disrupting effects of micro and nano plastic and their associated chemicals in mammals. Front. Endocrinol..

[cit151] Vietnam Investment Review , OXO-degradable plastics threaten sustainable development, 2023, cited 2025 Jan 14, available from: https://vir.com.vn/oxo-degradable-plastics-threaten-sustainable-development-100777.html

[cit152] Weber C. J., Hahn J., Opp C. (2022). Spatial connections between microplastics and heavy metal pollution within floodplain soils. Appl. Sci..

[cit153] Weber R., Herold C., Hollert H., Kamphues J., Blepp M., Ballschmiter K. (2018). Reviewing the relevance of dioxin and PCB sources for food from animal origin and the need for their inventory, control and management. Environ. Sci. Eur..

[cit154] Wei X., Bähr R. (2024). A comparative study of 3D printing with virgin and recycled polylactic acid filaments. CIRP J. Manuf. Sci. Technol..

[cit155] Woods M. N., Stack M. E., Fields D. M., Shaw S. D., Matrai P. A. (2018). Microplastic fiber uptake, ingestion, and egestion rates in the blue mussel (Mytilus edulis). Mar. Pollut. Bull..

[cit156] Salinas J., Carpena V., Martínez-Gallardo M. R., Segado M., Estrella-González M. J., Toribio A. J. (2023). *et al.*, Development of plastic-degrading microbial consortia by induced selection in microcosms. Front. Microbiol..

[cit157] Salinas J., Martínez-Gallardo M. R., Jurado M. M., Suárez-Estrella F., López-González J. A., Estrella-González M. J. (2024). *et al.*, Microbial consortia for multi-plastic waste biodegradation: selection and validation. Environ. Technol. Innov..

[cit158] Sciorio R., Tramontano L., Adel M., Fleming S. (2024). Decrease in sperm parameters in the 21st Century: obesity, lifestyle, or environmental factors? An updated narrative review. J. Pers. Med..

[cit159] Seid-Mohammadi A., Asgari G., Rafiee M., Samadi M. T., Nouri F., Pirsaheb M. (2022). *et al.*, Fate and inhibition of Bis (2-Ethylhexyl) phthalate in biophysical reactors for treating real landfill leachate. Process Saf. Environ. Prot..

[cit160] Sengupta D., Ilankoon I. M. S. K., Kang K. D., Chong M. N. (2023). Circular economy and household e-waste management in India. Part II: a case study on informal e-waste collectors (Kabadiwalas) in India. Miner. Eng..

[cit161] Sevilla M. E., Garcia M. D., Perez-Castillo Y., Armijos-Jaramillo V., Casado S., Vizuete K. (2023). *et al.*, Degradation of PET bottles by an engineered Ideonella sakaiensis PETase. Polymers.

[cit162] Palsania P., Singhal K., Dar M. A., Kaushik G. (2024). Food grade plastics and Bisphenol A: associated risks, toxicity, and bioremediation approaches. J. Hazard. Mater..

[cit163] Pandey P., Dhiman M., Kansal A., Subudhi S. P. (2023). Plastic waste management for sustainable environment: techniques and approaches. Waste Dispos. Sustain. Energy.

[cit164] Pantos O. (2022). Microplastics: impacts on corals and other reef organisms. Emerging Top. Life Sci..

[cit165] Paramasivam A., Murugan R., Jeraud M., Dakkumadugula A., Periyasamy R., Arjunan S. (2024). Additives in processed foods as a potential source of endocrine-disrupting chemicals: a review. J. Xenobiot..

[cit166] Pathak G., Nichter M., Hardon A., Moyer E., Latkar A., Simbaya J. (2023). *et al.*, Plastic pollution and the open burning of plastic wastes. Glob. Environ. Change.

[cit167] Paul S., Nath S., Bhattacharjee S., Mukherjee S. (2024). Unveiling the effects of microplastics pollution on marine fauna. Blue Biotechnol..

[cit168] Pinto Da Costa J. , Rocha Santos T. and Duarte A., The Environmental Impacts of Plastics and Micro-plastics Use, Waste and Pollution: EU and National Measures, Eur Union, 2020, pp. 10–62

[cit169] Plunk E. C., Richards S. M. (2020). Endocrine-disrupting air pollutants and their effects on the hypothalamus-pituitary-gonadal axis. Int. J. Mol. Sci..

[cit170] Qian Y., Shao H., Ying X., Huang W., Hua Y. (2020). The endocrine disruption of prenatal phthalate exposure in mother and offspring. Front. Public Heal..

[cit171] Rabiu M. K., Jaeger-Erben M. (2024). Reducing single-use plastic in everyday social practices: insights from a living lab experiment. Resour. Conserv. Recycl..

[cit172] Radke E. G., Braun J. M., Meeker J. D., Cooper G. S. (2018). Phthalate exposure and male reproductive outcomes: a systematic review of the human epidemiological evidence. Environ. Int..

[cit173] Ramadan B. S., Rosmalina R. T.-S.-M., Khair H., Rachman I., Matsumoto T. (2023). Potential risks of open waste burning at the household level: a case study of Semarang, Indonesia. Aerosol Air Qual. Res..

[cit174] Ramadan M., Cooper B., Posnack N. G. (2020). Bisphenols and phthalates: plastic chemical exposures can contribute to adverse cardiovascular health outcomes. Birth Defects Res. Res.

[cit175] Rani M. and Meenu S. U., Environmental Risk Assessment of Plastics and Its Additives, in Handbook of Green and Sustainable Nanotechnology, Cham, Springer International Publishing, 2023, pp. 2597–2622, 10.1007/978-3-031-16101-8_33

